# The association between substance use and subsequent employment among students: prospective findings from the CONSTANCES cohort

**DOI:** 10.1007/s00127-022-02357-0

**Published:** 2022-09-02

**Authors:** Rita El Haddad, Joane Matta, Cédric Lemogne, Maria Melchior, Marie Zins, Guillaume Airagnes

**Affiliations:** 1 INSERM, Population-Based Epidemiological Cohorts Unit, UMS 011, Hôpital Paul Brousse, Bâtiment 15/16, 16 avenue Paul Vaillant-Couturier, 94807 Villejuif, France; 2 AP-HP, Hôpital Hôtel-Dieu, DMU Psychiatrie et Addictologie, Service de Psychiatrie de l’adulte, INSERM, Institut de Psychiatrie et Neurosciences de Paris (IPNP), UMR_S1266, Université de Paris, Paris, France; 3 INSERM UMR_S 1136, Institut Pierre Louis d’Épidémiologie et de Santé Publique IPLESP, Sorbonne Université, Paris, France; 4 AP-HP, Hôpital Européen Georges Pompidou, DMU Psychiatrie et Addictologie, Centre Ambulatoire d’Addictologie, INSERM, Population-Based Epidemiological Cohorts Unit, Université de Paris, UMS 011, Villejuif, France

**Keywords:** Tobacco use, Cannabis use, Alcohol use, Employment, Youth

## Abstract

**Purpose:**

To examine prospectively associations between substance use and subsequent employment among young students.

**Methods:**

From the French population-based CONSTANCES cohort, 1427 students who never worked were included between 2012 and 2018 and followed up for 2.1 years on average. Generalized estimating equations computed the odds of being unemployed versus employed according to substance use at baseline controlling for sociodemographic factors and depressive state. Tobacco use (smoking status and number of cigarettes), cannabis use frequency, and at-risk alcohol use according to the Alcohol Use Disorder Identification Test (total score > 7) were introduced separately in the models.

**Results:**

Tobacco use was not significantly associated with employment. Cannabis use at least weekly was associated with increased odds of being unemployed OR 1.73 (1.16–2.57). At-risk alcohol use was no longer significantly associated with employment after adjustment for depressive state, while analyses on sub-scores of alcohol use suggested that alcohol dependence was associated with increased odds of being unemployed OR 1.65 (1.16–2.34).

**Conclusion:**

Public health campaigns targeting youth should include lower chances of getting employed among the detrimental roles of regular cannabis use and at-risk alcohol use.

**Supplementary Information:**

The online version contains supplementary material available at 10.1007/s00127-022-02357-0.

## Introduction

Substance use is a leading cause of premature death worldwide [1]. The most commonly used substances are tobacco, cannabis, and alcohol, and they are more likely to be initiated during adolescence and young adulthood [2]. In France, in 2017, among 18 to 25 years old, 35.3% of men and 29.2% of women were daily smokers, 27% were cannabis users, 80% were alcohol users and 13% reported binge drinking at least once per week [3]. Other than being significant financial burdens, substance use may result in serious consequences on physical and mental health, social well-being, educational achievements, and professional career/employment [4, 5]. On the other hand, youth unemployment is a global issue which was potentially aggravated by the Covid-19 pandemic [6, 7]. According to the International Labour Organization (ILO), prior to the Covid-19 crisis, youth were three times more likely to be unemployed compared to the general labor force [8]. France in particular has seen in the last decade higher rates of youth unemployment compared to other European Union countries [9]. Among the students who graduated in 2019, only 53.8% were employed within a year, and in 2021, the unemployment rate in young people aged from 15 to 24 years was of 19.0% compared to 7.9% for the entire labor force [10, 11]. For instance, in the same year, these figures were of 5.8% and 3.1% in Germany [12]. Transitioning from education to employment can be particularly challenging even though it may be easier for highly educated individuals to find a job [6, 13–15]. Youth unemployment has been reported to be associated with severe detrimental consequences on physical and mental health including depression, and substance use [16–18]. However, even if higher prevalence of substance use was reported in unemployed young adults compared to their employed peers [19], it remains unclear whether substance use in youth could be associated with a lower likelihood of accessing employment.

Substance use is associated with poorer neuropsychological functioning especially during adolescence and young adulthood, a crucial period for neurodevelopment [20]. Tobacco use can be easily addictive and an increase of stress and anxiety, which could have a negative impact during a job interview or a trial period, is commonly associated with the need for nicotine [21]. Cannabis and alcohol use adversely affect cognitive functioning (e.g., inhibitory control, memory and attentional capacities), which may hinder the job searching and the chances to be recruited [20]. 

Among the few studies that examined the role of substance use on employment in youth, most of them found significant associations [5, 22–24], whereas others did not [25]. For instance, a longitudinal study reported that among young Swiss men who were neither in training nor in employment, daily tobacco use and cannabis use at baseline were associated with an increased risk of remaining unemployed at follow-up [26]. In addition, a longitudinal study in a New Zealand birth cohort revealed that high cannabis use during adolescence and young adulthood was associated with higher unemployment in the mid-twenties [24]. In a study conducted in the United States, there was no association between alcohol use and full-time employment among students. The frequency of heavy episodic drinking was, however, negatively associated with the odds of full-time employment [23]. None of the prior studies regarding alcohol use distinguished between frequency of use and level of dependence. To the best of our knowledge, no study examined prospectively the role of tobacco, cannabis, and alcohol use on employment status in the same population of students or trainees, especially that no study focused as well on the transition from education to employment.

We took advantage of the national population-based CONSTANCES cohort, a large randomized sample of the French population from different sociodemographic backgrounds, including students who had never been employed [27]. The aim of this study was to examine prospectively the associations between substance use (i.e., tobacco, cannabis, and alcohol) and employment status among students participating in the CONSTANCES cohort while taking into consideration their sociodemographic factors and depressive state. We hypothesized that young people who are daily smokers, regular cannabis users and at-risk alcohol users will be more likely to be unemployed at follow-up compared to non-smokers, no cannabis users and alcohol users at lower risk, respectively, after adjustment for sociodemographic factors and depressive state.

## Methodology

### Population

**Fig. 1:**
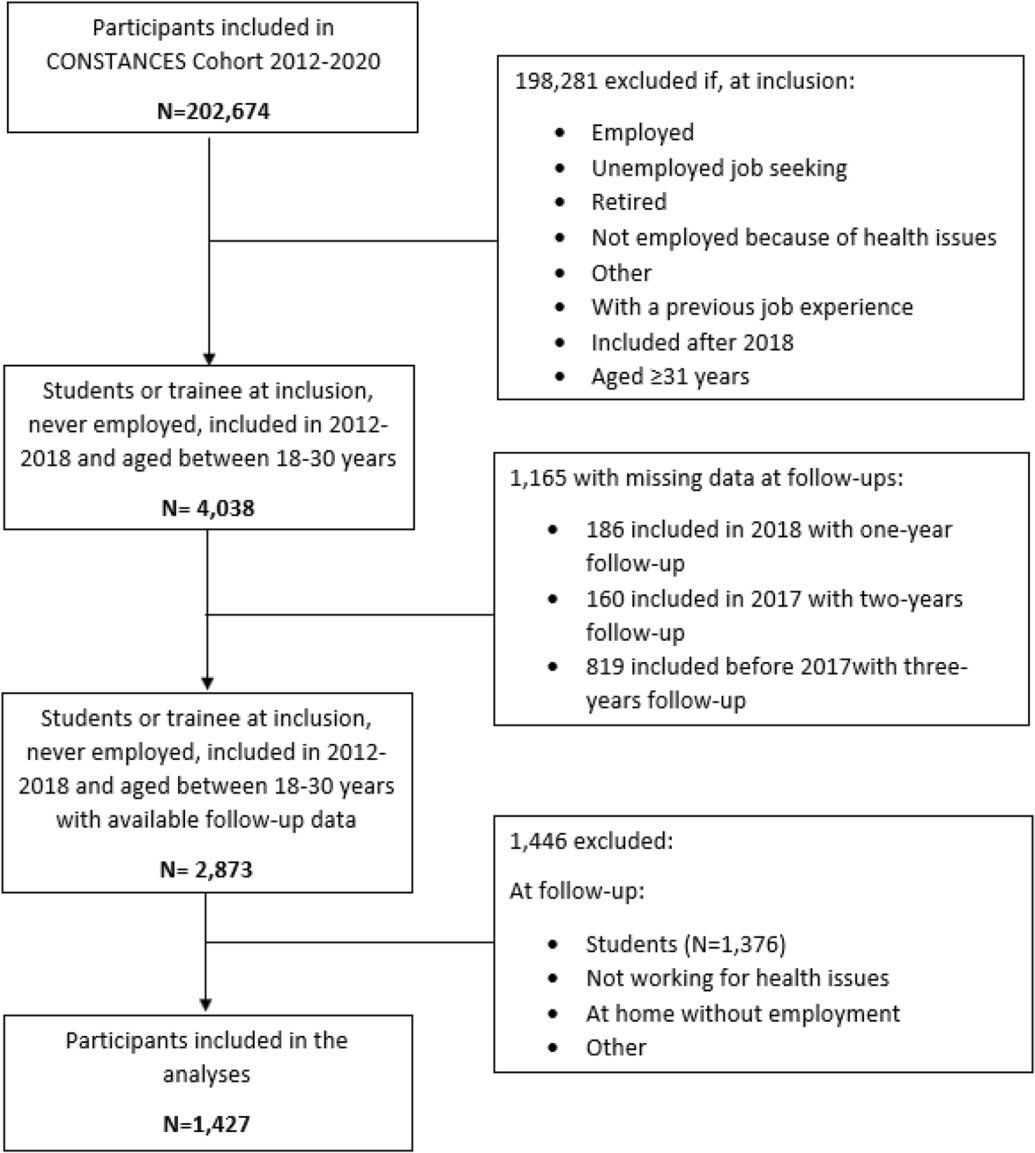
Flow chart of population selection

The data for this study were derived from the CONSTANCES cohort, a large national population-based cohort with a randomized sample of adults aged between 18 and 69 at inclusion (93,905 (46.3%) men and 108,769 (53.7%) women in 2020). The methodology of the CONSTANCES cohort study has already been detailed elsewhere [27]. Individuals aged 18–69, affiliated to the main national health insurance covering around 85% of the population, were randomly chosen to participate in the study according to an unequal probability sampling stratified by gender, age, social category and area. At enrollment, participants completed a self-administered questionnaire and underwent a health examination in one of the 22 selected health screening centers located in 20 ‘départements’ in the principal regions of France. An ongoing follow-up includes annual self-administered questionnaires, health examinations every 4 years, and passive data collection by linkage to the two national databases National Health Insurance Fund and National Pension Insurance Fund. The population studied here was limited to students or interns included between 2012 and 2018. Among these participants, we only selected those who reported to have never been employed throughout their lifetime. Then, we excluded those who did not have a minimum of 1 year of follow-up data available. We included participants aged between 18 and 30 years because we consider that not having the possibility to find a job above this age may indicate unusual health and/or social impairment compared to the general population [28]. Among these 4038 participants, 1165 did not report their employment status during the follow-ups (186 participants included in 2018 and thus with only 1-year follow-up, 160 included in 2017 and thus with only 2-year follow-up and 819 included between 2012 and 2016 and thus with 3-year follow-up), 1376 participants reported being students at follow-up and 70 participants only reported not working for health reasons or being at home without employment or any other situation at follow-up. Therefore, 1427 participants were included in the statistical analyses (49 had 1 year of follow-up, 153 had 2 years of follow-up and 1225 had 3 years of follow-up) (Fig. 1).

### Research ethics approval

The CONSTANCES cohort has obtained the authorization of the National Data Protection Authority (Commission Nationale de l’Informatique et des Libertés, no. 910486) and was approved by the Institutional Review Board of the National Institute for Medical Research—INSERM (no. 01-011). All participants have provided a written informed consent.

### Substance use at baseline

#### Tobacco use

At baseline, participants’ smoking status was self-reported as follows: never smoker, former smoker or current smoker. For current smokers, the number of cigarettes per day was collected in order to assess daily tobacco consumption. From the latter two variables, tobacco use was computed as a categorical variable as such: (1) never smokers, (2) former smokers, (3) current light smokers (1–9 cigarettes per day), (4) current moderate smokers (10–19 cigarettes per day), and (5) current heavy smokers (> 19 cigarettes per day) [29]. 

#### Cannabis use

The frequency of cannabis consumption was self-reported at baseline by answering the following questions: “Have you ever consumed cannabis? If yes, over the past 12 months, have you taken cannabis? Over the past 30 days, have you taken cannabis?”. One categorical variable was computed from the previous questions as follows: (1) never used; (2) no consumption during the last 12 months; (3) at least once during the last 12 months but less than once a month; (4) at least once a month but less than once per week; (5) once per week or more.

#### Alcohol use

To evaluate alcohol use at baseline, we used the French version of the Alcohol Use Disorders Identification Test (AUDIT) that includes 10 items [30]. The total AUDIT score was calculated by adding the scores of all ten items and was categorized as: (1) no use (a score of 0), (2) low risk (1–7), and (3) at-risk (> 7). We chose “low risk alcohol use” as the reference category because contrary to tobacco and cannabis, for alcohol, international guidelines consider that there is a consumption at lower risk since the damages are mainly dose dependent and exponential [1]. In addition, alcohol use at lower risk corresponds to the most commonly pattern of alcohol use in the general population whereas both at-risk alcohol use and non-alcohol use correspond to unusual patterns of use, at least in European countries [31]. Regarding non-alcohol users, previous studies have shown that alcohol abstinence is associated with poor health status which contraindicates alcohol use, and at least for some people because of the consequences of previous excessive use [32].

In order to differentiate between the frequency of alcohol use and alcohol dependence, two sub-scores were computed: one sub-score for “frequency of use” by adding the scores of the first 3 items, and one sub-score for “alcohol dependence” by adding the score of items 4 to 10 of the AUDIT [33]. They were then analyzed as tertiles.

### Employment status at follow-up

Employment status was self-reported annually over a 3-year period of follow-up with the following question: “What is your current employment situation?”: (1) employed, including on sick leave, leave without pay or availability, maternity/paternity/adoption/parental leave; (2) job seeker; (3) retired or withdrawn from business; (4) in training (high school student, student, trainee, apprentice or other; (5) does not work for health reasons; (6) at home without employment; (7) other situation. At follow-up, participants who answered “1” were considered as “employed”. Since individuals who are not seeking a job may have different profiles than the ones who are, we only kept those who selected option (2) in the “unemployed” category. As expected, since our sample is limited to young adults, no participants selected option (3). The other categories were thus not included in the main analyses.

### Covariates at baseline

We used the following sociodemographic variables: age, gender, education, area deprivation index score, and living place were self-reported at baseline. Age was modeled as a continuous variable. Education was based on the International Standard Classification of Education (ISCED 2011) and analyzed as a continuous variable [34]. The area deprivation index was used as a continuous variable to represent spatial socioeconomic disparities, including four components: household income, percentage of high school graduates, percentage of blue-collar workers and unemployment rate [35]. Participants who answered living with others and at least with one ascending line relatives were considered as living with their parents.

Depressive symptoms were measured at baseline with the Center of Epidemiologic Studies Depression scale (CESD). The CESD score was dichotomized: a score ≥ 19 was considered as indicating a clinically significant depressive state (sensitivity/specificity for the diagnosis of major depression: 0.85/0.86) [36].

### Statistical analysis

In line with our a priori hypotheses, we aimed to estimate the odds of unemployment at follow-up according to substance use at baseline over a 3-year follow-up period. Thus, we conducted generalized estimating equations (GEE) since this type of analysis is able to take into consideration participants who had 1, 2 or 3 years of follow-up. In the main analyses, the substances were introduced in separate models. Entries where participants declared being students at follow-up were discarded. Consequently “employment status” was modeled as a binary variable with two option being ‘employed’ (the reference category) versus ‘unemployed’.

For each substance, three models of adjustments were considered as follows: model 1 represents the crude association between each substance and employment status, model two further adjusted for sociodemographic factors, and model three additionally adjusted for depressive state.

#### Exploratory analyses

1. To examine whether the use of several substances that were found to be significantly associated with employment could be associated with a greater likelihood of unemployment compared to the use of a single substance, we used a categorical variable distinguishing between single and multiple substance use.
2. Interactions between the substances and all the covariables were tested to search for potential moderating effects. In case of a significant interaction, stratified analyses were performed.

#### Sensitivity analyses

We conducted several sensitivity analyses as follows:

1. Parents’ highest occupational grade ((1) home or other; (2) blue-collar worker, craftsman, farmer or employee; (3) intermediate worker; (4) executive, higher intellectual profession) was added to the models as an indicator of the participants’ social background and the potential support, such as financial, they may receive. Since parents’ occupational grade may be highly correlated with other covariates, it was added only as an addition to prevent from over adjustment.
2. The three substances were included in the same model to see if a substance prevails over others.
3. Analyses were re-conducted after adding to the unemployed category participants who reported not working for health reasons, at home without employment and other situations at follow-up to evaluate the odds of all forms of unemployment compared to being employed.
4. Analyses were re-conducted after excluding participants with psychiatric history (depression, suicide attempt or other diagnosed psychiatric disorders).
5. Analyses were re-conducted after excluding participants who initiated alcohol use at an early age (i.e. < 13 years, roughly corresponding to the first decile of age in our sample) since early initiation could indicate particular vulnerability to both substances use and social impairment.

Finally, by computing e-values, we aimed to estimate the minimal strength of association that an unmeasured confounder (or a set of unmeasured confounders) should have with both substance use and employment status to fully explain the reported associations [37]

Among the included participants (*N* = 4038), prevalence of missing data regarding the independent variables ranged from 1.5% for education to 14.3% for the AUDIT total score with an average of 3.2%. These missing data were handled by multiple imputation in 10 datasets. The results of the combined 10 datasets were reported [38]. Missing data regarding the outcome (i.e., employment at follow-up) were not imputed and attrition was described in descriptive analyses.

Statistical significance was determined using a two-sided alpha set at 0.05. Analyses were carried out using SPSS IBM Statistics for Windows version 21.

## Results

### Participants’ characteristics

The characteristics of the 4038 participants are presented in Table 1. The included participants were followed up for 2.1 (0.8) years in average. Among the, 1427 included participants, 22.8% were current smokers, 9.1% consumed cannabis once per week or more and 26.1% were at-risk alcohol users. After 1, 2 and 3 years of follow-up, 562 (45.2%), 671 (59.8%), and 742 (80.4%) participants, respectively, found a job (supplemental Table 1). The characteristics of the participants according to the missing data for employment status at follow-up and for each duration of follow-up (i.e., from 1 to 3 years) are presented in Supplemental Table 2. However, these participants had not answered the entire questionnaire and not specifically the question about their employment status.

**Table 1:** Characteristics of the 4038 participants included at baseline and according to the employment status over a 3 years of follow-up

	Total	Included participants			Student^c^	Other	Missing^d^
		Total	Employed^a^	Unemployed^b^			
Categorical variables	4038 (100) *N* (%)	1427 (100) *N* (%)	1242 (87.0) *N* (%)	185 (13.0) *N* (%)	1376 (34.1) *N* (%)	70 (1.7) *N* (%)	1165 (28.9) *N* (%)
Tobacco use							
Non-smoker	2964 (66.7)	964 (67.5)	850 (68.4)	113 (61.1)	991 (72.0)	42 (60.0)	698 (59.9)
Former smoker	391 (9.7)	138 (9.7)	118 (9.5)	20 (10.8)	131 (9.5)	9 (12.9)	113 (9.7)
Current light smoker	643 (15.9)	212 (14.8)	176 (14.2)	36 (19.4)	191 (13.9)	14 (20.0)	226 (19.4)
Current moderate Smoker	273 (6.7)	103 (7.2)	91 (7.3)	12 (6.5)	53 (3.9)	5 (7.1)	112 (9.6)
Current heavy smoker	37 (1)	11 (0.8)	7 (0.6)	4 (2.2)	10 (0.7)	0 (0.0)	16 (1.4)
Cannabis consumption							
Never used	1889 (46.8)	686 (48.1)	606 (48.8)	80 (43.2)	687 (49.9)	25 (35.7)	491 (42.2)
No consumption during the last 12 months	801 (19.8)	310 (21.7)	274 (22.0)	36 (19.5)	242 (17.6)	17 (24.3)	232 (19.9)
At least once during the last 12 months but less than once a month	574 (14.2)	186 (13.0)	162 (13.0)	24 (13.0)	223 (16.2)	10 (14.3)	155 (13.3)
At least once a month but less than once per week	332 (8.2)	115 (8.1)	100 (8.1)	15 (8.1)	104 (7.6)	8 (11.4)	105 (9.0)
Once per week or more	442 (11.0)	130 (9.1)	100 (8.1)	30 (16.2)	120 (8.7)	10 (14.3)	182 (15.6)
AUDIT Score^e^							
0	274 (6.8)	70 (4.9)	55 (4.4)	15 (8.1)	78 (5.7)	3 (4.3)	123 (10.6)
1–7	2623 (64.9)	983 (69.0)	871 (70.1)	112 (60.5)	920 (66.9)	41 (58.6)	678 (58.2)
> 7	1141 (28.3)	373 (26.1)	316 (25.5)	58 (31.4)	378 (27.4)	26 (37.1)	364 (31.2)
Alcohol frequency of use^f^							
[0–2]	1329 (32.9)	464 (32.5)	400 (32.2)	64 (34.6)	468 (34.0)	20 (28.5)	377 (32.4)
[3–5]	1184 (29.3)	535 (37.5)	474 (38.2)	61 (33.0)	511 (37.1)	22 (31.5)	422 (36.2)
[6 +]	916 (22.7)	428 (30.0)	368 (29.6)	60 (32.4)	397 (28.9)	28 (40.0)	366 (31.4)
Alcohol dependence^g^							
0	1938 (48.0)	703 (49.3)	618 (49.8)	85 (46.0)	672 (48.8)	28 (40.0)	535 (45.9)
[1, 2]	1184 (29.3)	435 (30.5)	380 (30.6)	55 (29.7)	398 (28.9)	24 (34.3)	327 (28.1)
[3 +]	916 (22.7)	289 (20.2)	244 (19.6)	45 (24.3)	306 (22.2)	18 (25.7)	303 (26.0)
Gender							
Men	1481 (36.7)	455 (31.9)	396 (31.9)	59 (31.9)	514 (37.4)	31 (44.3)	481 (41.5)
Women	2557 (63.3)	972 (68.1)	846 (68.1)	126 (68.1)	862 (62.6)	39 (55.7)	684 (58.7)
Education^h^							
Early childhood or primary	21 (0.5)	5 (0.3)	4 (0.3)	1 (0.5)	7 (0.5)	2 (2.9)	7 (0.6)
Lower secondary	130 (3.2)	29 (2.0)	17 (1.4)	12 (6.5)	52 (3.8)	1 (1.4)	48 (4.1)
Upper secondary or post-secondary non-tertiary	2235 (55.3)	652 (45.7)	562 (45.2)	90 (48.5)	929 (67.5)	37 (52.9)	617 (53.0)
Short-cycle tertiary or bachelor’s	1246 (30.9)	535 (37.5)	469 (37.8)	66 (35.7)	335 (24.3)	20 (28.5)	356 (30.6)
Master’s or doctoral	406 (10.1)	206 (14.5)	190 (15.3)	16 (8.6)	53 (3.9)	10 (14.3)	137 (11.7)
Living place							
With parents	2116 (52.4)	718 (50.3)	615 (49.5)	103 (55.7)	820 (59.6)	31 (44.3)	547 (47.0)
Others	1922 (47.6)	709 (49.7)	627 (50.5)	82 (44.3)	556 (40.4)	39 (55.7)	618 (53.0)
Depressive state^i^							
No	3217 (79.7)	1174 (82.3)	1051 (84.6)	123 (66.5)	1123 (81.6)	51 (72.9)	869 (74.6)
Yes	821 (20.3)	253 (17.7)	191 (15.4)	62 (33.5)	253 (18.4)	19 (27.1)	296 (25.4)

Continuous variables	Mean (SD)	Mean (SD)	Mean (SD)	Mean (SD)	Mean (SD)	Mean (SD)	Mean (SD)
Age (years)	21.4 (2.4)	21.9 (2.3)	22.0 (2.4)	21.8 (2.3)	20.5 (1.8)	21.5 (2.4)	21.7 (2.4)
Area deprivation index^j^	− 0.9 (1.7)	− 0.9 (1.7)	− 0.9 (1.7)	− 0.8 (1.5)	− 0.9 (1.7)	0.7 (1.6)	− 0.8 (1.7)

^a^Participants who replied “employed” at least once over the 3 years of follow-up

^b^Participants who replied at least once “unemployed job seeking” over the 3 years of follow-up and never “employed”

^c^Participants who only replied “student” over the 3 years of follow-up

^d^Participants who never reported their employment status over the 3 years of follow-up

^e^Alcohol Use Disorders Identification Test to evaluate alcohol use

^f^AUDIT sub-score for frequency of use by adding the scores of the first 3 items

^g^AUDIT sub-score for alcohol dependence by adding the score of items 4, 5, 6, 7, 8, 9 and 10

^h^Based on the International Standard Classification of Education

^i^Measured using the Center of Epidemiologic Studies Depression scale (CESD) and a score ≥ 19

^j^Representing spatial socioeconomic disparities

### Tobacco use and employment status

No significant association between tobacco use and accessing employment were found in the univariable or multivariable analyses. Even though estimates were higher for current heavy smokers than non-smokers in univariable analysis, after adjustment for sociodemographic factors and after further adjustment for depressive state, associations were not statistically significant (OR 2.66, 95% CI 0.80 to 8.75; OR 2.60, 95% CI 0.86 to 7.84; and OR 1.78, 95% CI 0.58 to 5.41, respectively) (Table 2).

**Table 2:** Association between tobacco use and employment status over 3 years of follow-up (*n* = 1427)

Being unemployed over 3 years of follow-up									
	Model 1^a^			Model 2^b^			Model 3^c^		
	OR	95% CI		OR	95% CI		OR	95% CI	
Tobacco use									
Non-smoker	Ref	–	–	Ref	–	–	Ref	–	–
Former smoker	1.10	0.73	1.64	1.07	0.71	1.62	1.00	0.66	1.50
Current light smoker	1.20	0.85	1.70	1.18	0.83	1.67	1.06	0.74	1.52
Current moderate smoker	0.99	0.58	1.69	0.94	0.55	1.63	0.86	0.49	1.51
Current heavy smoker	2.66	0.80	8.75	2.60	0.86	7.84	1.78	0.58	5.41
Years of follow-up	0.74	0.66	0.85	0.75	0.66	0.85	0.75	0.66	0.86
Age				1.10	1.03	1.17	1.08	1.01	1.16
Gender									
Men				Ref	–	–	Ref	–	–
Women				0.93	0.72	1.21	0.85	0.66	1.10
Education level^d^				0.60	0.48	0.73	0.63	0.51	0.77
Area deprivation index^e^				0.99	0.92	1.07	1.01	0.92	1.06
Living place									
With parents				Ref	–	–	Ref	–	–
Other				0.84	0.66	1.09	0.85	0.66	1.09
Depressive state^f^									
No							Ref	–	–
Yes							2.13	1.58	2.86

^a^Univariate analysis adjusted to years of follow-up

^b^Adjusted for sociodemographic factors

^c^Adjusted for sociodemographic factors and depressive state

^d^Based on the International Standard Classification of Education

^e^Representing spatial socioeconomic disparities

^f^Measured using the Center of Epidemiologic Studies Depression scale (CESD) and a score ≥ 19

### Cannabis use and employment status

In univariable analyses, using cannabis once per week or more was associated with a significant increased odd of being unemployed at follow-up compared to not consuming cannabis (OR 1.94, 95% CI 1.29 to 2.90). This association persisted after adjustment for sociodemographic factors (OR 1.94, 95% CI 1.30 to 2.89) and for depressive state (OR 1.73, 95% CI 1.16 to 2.57) (Table 3).

**Table 3:** Association between cannabis use and employment status over 3 years of follow-up (*n* = 1427)

Being unemployed over 3 years of follow-up									
	Model 1^a^			Model 2^b^			Model 3^c^		
	OR	95% CI		OR	95% CI		OR	95% CI	
Cannabis consumption									
Never used	Ref	–	–	Ref	–	–	Ref	–	–
Not during the previous year	0.81	0.59	1.11	0.84	0.61	1.15	0.80	0.58	1.11
At least once during the last 12 months but less than once a month	1.10	0.75	1.59	1.16	0.80	1.69	1.08	0.74	1.59
At least once a month but less than once per week	1.04	0.66	1.66	1.12	0.69	1.80	1.06	0.66	1.71
Once per week or more	1.94	1.29	2.90	1.94	1.30	2.89	1.73	1.16	2.57
Years of follow-up	0.74	0.65	0.85	0.75	0.66	0.85	0.75	0.66	0.86
Age				1.11	1.04	1.19	1.09	1.02	1.17
Gender									
Men				Ref	–	–	Ref	–	–
Women				0.99	0.77	1.29	0.90	0.69	1.17
Education level^d^				0.60	0.49	0.74	0.63	0.52	0.78
Area Deprivation index^e^				1.00	0.93	1.07	0.99	0.93	1.07
Living place									
With parents				Ref	–	–	Ref	–	–
Other				0.83	0.65	1.07	0.84	0.65	1.08
Depressive state^f^									
No							Ref	–	–
Yes							2.08	1.55	2.80

^a^Univariate analysis adjusted to years of follow-up

^b^Adjusted for sociodemographic factors

^c^Adjusted for sociodemographic factors and depressive state

^d^Based on the International Standard Classification of Education

^e^Representing spatial socioeconomic disparities

^f^Measured using the Center of Epidemiologic Studies Depression scale (CESD) and a score ≥ 19

### Alcohol use and employment status

In univariable analyses, not consuming alcohol or being in an at-risk alcohol use category was associated with a significant increased odd of being unemployed at follow-up compared to a low-risk alcohol use (OR 1.85, 95% CI 1.12–3.07 and OR 1.31, 95% CI 1.01–1.72, respectively). After adjusting for sociodemographic factors, only the association with at-risk alcohol use persisted (OR 1.35, 95% CI 1.02–1.78), but this association did not persist after adjusting for depressive state (OR 1.31, 95% CI 0.99–1.72) (Table 4).

**Table 4:** Association between alcohol use and employment status over a 3 years of follow-up (*n* = 1427)

Being unemployed over 3 years of follow-up									
	Model 1^a^			Model 2^b^			Model 3^c^		
	OR	95% CI		OR	95% CI		OR	95% CI	
AUDIT^d^									
Low risk	Ref	–	–	Ref	–	–	Ref	–	–
No use	1.85	1.12	3.07	1.57	0.93	2.64	1.50	0.88	2.53
At risk	1.31	1.01	1.72	1.35	1.02	1.78	1.31	0.99	1.72
Years of follow-up	0.74	0.65	0.84	0.75	0.65	0.85	0.75	0.66	0.86
Age				1.10	1.02	1.17	1.08	1.01	1.15
Gender									
Men				Ref	–	–	Ref	–	–
Women				0.99	0.76	1.29	0.90	0.69	1.17
Education level^e^				0.60	0.49	0.74	0.64	0.52	0.78
Area deprivation index^f^				0.99	0.92	1.07	0.99	0.92	1.06
Living place									
With parents				Ref	–	–	Ref	–	–
Other				0.84	0.65	1.08	0.84	0.65	1.08
Depressive state^g^									
No							Ref	–	–
Yes							2.12	1.58	2.84

^a^Univariate analysis adjusted to years of follow-up

^b^Adjusted for sociodemographic factors

^c^Adjusted for sociodemographic factors and depressive state

^d^Alcohol Use Disorders Identification Test to evaluate alcohol use

^e^Based on the International Standard Classification of Education

^f^Representing spatial socioeconomic disparities

^g^Measured using the Center of Epidemiologic Studies Depression scale (CESD) and a score ≥ 19

In univariable analyses, being in the highest tertile of alcohol dependency compared to the lowest was significantly associated with an increased odd of being unemployed (OR 1.80, 95% CI 1.27–2.54). This association persisted after adjusting for sociodemographic factors (OR 1.74, 95% CI 1.23–2.46) and depressive state (OR 1.65, 95% CI 1.16–2.34) (Table 5). Frequency of alcohol use was not significantly associated with employment.

**Table 5:** Association between alcohol use and employment status over a 3 years of follow-up by differentiating between frequency of use and dependence (n = 1427)

Being unemployed over 3 years of follow-up									
	Model 1^a^			Model 2^b^			Model 3^c^		
	OR	95% CI		OR	95% CI		OR	95% CI	
Alcohol frequency of use^d^									
[0–2]	Ref	–	–	Ref	–	–	Ref	–	–
[3–5]	0.82	0.59	1.12	0.87	0.63	1.21	0.84	0.60	1.17
[6 +]	0.74	0.52	1.05	0.84	0.98	1.20	0.82	0.57	1.16
Alcohol dependence^e^									
[0]	Ref	–	–	Ref	–	–	Ref	–	–
[1, 2]	1.24	0.91	1.69	1.21	0.89	1.64	1.18	0.86	1.60
[3 +]	1.80	1.27	2.54	1.74	1.23	2.46	1.65	1.16	2.34
Years of follow-up	0.75	0.66	0.85	0.75	0.66	0.86	0.76	0.66	0.87
Age				1.10	1.03	1.17	1.08	1.01	1.15
Gender									
Men				Ref	–	–	Ref	–	–
Women				0.96	0.73	1.25	0.87	0.66	1.13
Education level^f^				0.60	0.49	0.74	0.63	0.52	0.78
Area deprivation index^g^				0.99	0.92	1.07	0.99	0.92	1.07
Living place									
With parents				Ref	–	–	Ref	–	–
Other				0.85	0.66	1.09	0.85	0.66	1.09
Depressive state^h^									
No							Ref	–	–
Yes							2.11	1.58	2.83

^a^Univariate analysis adjusted to years of follow-up

^b^Adjusted for sociodemographic factors

^c^Adjusted for sociodemographic factors and depressive state

^d^AUDIT sub-score for frequency of use by adding the scores of the first 3 items

^e^AUDIT sub-score for alcohol dependence by adding the score of items 4, 5, 6, 7, 8, 9 and 10

^f^Based on the International Standard Classification of Education

^g^Representing spatial socioeconomic disparities

^h^Measured using the Center of Epidemiologic Studies Depression scale (CESD) and a score ≥ 19

### Comparison between single and multiple substance use

When cannabis use once per week or more and alcohol dependence were considered together, i.e., (1) consuming either one and (2) consuming both, the odd for cannabis use or alcohol use only was of 1.43 (95% CI 1.05 to 1.93) and the odd for the use of both substances was of 2.44 (95% CI 1.44–4.12) (*Z* score = 0.32; *p* = 0.374) (Table 6). In sensitivity analyses, there was an interaction between cannabis use and education. After stratification (less than a bachelor degree and bachelor degree or higher), cannabis use remained significantly associated with employment (supplemental Table 3). When parents’ highest occupational grade was added to the models, all associations remained unchanged (supplemental Tables 4–7). After adding the three substances in the same model, all associations remained significant for cannabis use and alcohol dependence (supplemental Table 8). After adding to the unemployed category participants who reported not working for health reasons, being at home without employment and any other situation at follow-up, the associations for cannabis use and alcohol dependence persisted, as well as for at-risk alcohol use which remained significantly associated to employment status after adjustment for sociodemographic factors and depressive state (supplemental Tables 9–12). After excluding participants with psychiatric history, all associations remained significant (supplemental Tables 13, 14). After excluding participants who initiated alcohol use at an early age, similar associations were found for alcohol use (supplemental Table 15).

**Table 6:** Association between alcohol dependence and cannabis use once per week or more with employment status over 3-year follow-up (*n* = 1427)

Being unemployed over 3 years of follow-up									
	Model 1^a^			Model 2^b^			Model 3^c^		
	OR	95% CI		OR	95% CI		OR	95% CI	
Substance use									
Alcohol dependence^d^ score < 3 and cannabis use less than once per week	Ref	–	–	Ref	–	–	Ref	–	–
Alcohol dependence score ≥ 3 or cannabis use once per week or more	1.47	1.09	1.98	1.48	1.10	2.01	1.43	1.05	1.93
Alcohol dependence score ≥ 3 and cannabis use once per week or more	2.74	1.56	4.80	2.75	1.60	4.72	2.44	1.44	4.12
Years of follow-up				0.76	0.66	0.86	0.76	0.67	0.87
Age				1.10	1.03	1.18	1.09	1.02	1.16
Gender									
Men				Ref	–	–	Ref	–	–
Women				1.01	0.78	1.31	0.91	0.71	1.19
Education level^e^				0.60	0.49	0.74	0.63	0.51	0.78
Area deprivation index^f^				1.01	0.93	1.08	1.01	0.93	1.08
Living place									
With parents				Ref	–	–	Ref	–	–
Other				0.83	0.65	1.07	0.84	0.65	1.08
Depressive state^g^									
No							Ref	–	–
Yes							2.05	1.53	2.75

^a^Univariate analysis adjusted to years of follow-up

^b^Adjusted for sociodemographic factors

^c^Adjusted for sociodemographic factors and depressive state

^d^AUDIT sub-score for alcohol dependence by adding the score of items 4, 5, 6, 7, 8, 9 and 10

^e^Based on the International Standard Classification of Education

^f^Representing spatial socioeconomic disparities

^g^Measured using the Center of Epidemiologic Studies Depression scale (CESD) and a score ≥ 19

Finally, a confounder or set of confounders would have to have an OR of 3.1-fold in the increase risk of unemployment and must be 2.85 times more prevalent in cannabis use to explain the observed odds ratio for cannabis use. This e-value was of 2.68 for alcohol dependence (Tables 7, 8, 9, 10, 11, 12).

**Table 7:** Characteristics of the 4038 participants included at baseline and according to the employment status over a 3 years of follow-up

	Total	Included participants			Student^c^	Other	Missing^d^
		Total	Employed^a^	Unemployed^b^			
Categorical variables	4038 (100) *N* (%)	1427 (100) *N* (%)	1242 (87.0) *N* (%)	185 (13.0) *N* (%)	1376 (34.1) *N* (%)	70 (1.7) *N* (%)	1165 (28.9) *N* (%)
Tobacco use							
Non-smoker	2964 (66.7)	964 (67.5)	850 (68.4)	113 (61.1)	991 (72.0)	42 (60.0)	698 (59.9)
Former smoker	391 (9.7)	138 (9.7)	118 (9.5)	20 (10.8)	131 (9.5)	9 (12.9)	113 (9.7)
Current light smoker	643 (15.9)	212 (14.8)	176 (14.2)	36 (19.4)	191 (13.9)	14 (20.0)	226 (19.4)
Current moderate smoker	273 (6.7)	103 (7.2)	91 (7.3)	12 (6.5)	53 (3.9)	5 (7.1)	112 (9.6)
Current heavy smoker	37 (1)	11 (0.8)	7 (0.6)	4 (2.2)	10 (0.7)	0 (0.0)	16 (1.4)
Cannabis consumption							
Never used	1889 (46.8)	686 (48.1)	606 (48.8)	80 (43.2)	687 (49.9)	25 (35.7)	491 (42.2)
No consumption during the last 12 months	801 (19.8)	310 (21.7)	274 (22.0)	36 (19.5)	242 (17.6)	17 (24.3)	232 (19.9)
At least once during the last 12 months but less than once a month	574 (14.2)	186 (13.0)	162 (13.0)	24 (13.0)	223 (16.2)	10 (14.3)	155 (13.3)
At least once a month but less than once per week	332 (8.2)	115 (8.1)	100 (8.1)	15 (8.1)	104 (7.6)	8 (11.4)	105 (9.0)
Once per week or more	442 (11.0)	130 (9.1)	100 (8.1)	30 (16.2)	120 (8.7)	10 (14.3)	182 (15.6)
AUDIT score^e^							
0	274 (6.8)	70 (4.9)	55 (4.4)	15 (8.1)	78 (5.7)	3 (4.3)	123 (10.6)
1–7	2623 (64.9)	983 (69.0)	871 (70.1)	112 (60.5)	920 (66.9)	41 (58.6)	678 (58.2)
> 7	1141 (28.3)	373 (26.1)	316 (25.5)	58 (31.4)	378 (27.4)	26 (37.1)	364 (31.2)
Alcohol frequency of use^f^							
[0–2]	1329 (32.9)	464 (32.5)	400 (32.2)	64 (34.6)	468 (34.0)	20 (28.5)	377 (32.4)
[3–5]	1184 (29.3)	535 (37.5)	474 (38.2)	61 (33.0)	511 (37.1)	22 (31.5)	422 (36.2)
[6 +]	916 (22.7)	428 (30.0)	368 (29.6)	60 (32.4)	397 (28.9)	28 (40.0)	366 (31.4)
Alcohol dependence^g^							
0	1938 (48.0)	703 (49.3)	618 (49.8)	85 (46.0)	672 (48.8)	28 (40.0)	535 (45.9)
[1, 2]	1184 (29.3)	435 (30.5)	380 (30.6)	55 (29.7)	398 (28.9)	24 (34.3)	327 (28.1)
[3 +]	916 (22.7)	289 (20.2)	244 (19.6)	45 (24.3)	306 (22.2)	18 (25.7)	303 (26.0)
Gender							
Men	1481 (36.7)	455 (31.9)	396 (31.9)	59 (31.9)	514 (37.4)	31 (44.3)	481 (41.5)
Women	2557 (63.3)	972 (68.1)	846 (68.1)	126 (68.1)	862 (62.6)	39 (55.7)	684 (58.7)
Education^h^							
Early childhood or primary	21 (0.5)	5 (0.3)	4 (0.3)	1 (0.5)	7 (0.5)	2 (2.9)	7 (0.6)
Lower secondary	130 (3.2)	29 (2.0)	17 (1.4)	12 (6.5)	52 (3.8)	1 (1.4)	48 (4.1)
Upper secondary or post-secondary non-tertiary	2235 (55.3)	652 (45.7)	562 (45.2)	90 (48.5)	929 (67.5)	37 (52.9)	617 (53.0)
Short-cycle tertiary or bachelor’s	1246 (30.9)	535 (37.5)	469 (37.8)	66 (35.7)	335 (24.3)	20 (28.5)	356 (30.6)
Master’s or doctoral	406 (10.1)	206 (14.5)	190 (15.3)	16 (8.6)	53 (3.9)	10 (14.3)	137 (11.7)
Living place							
With parents	2116 (52.4)	718 (50.3)	615 (49.5)	103 (55.7)	820 (59.6)	31 (44.3)	547 (47.0)
Others	1922 (47.6)	709 (49.7)	627 (50.5)	82 (44.3)	556 (40.4)	39 (55.7)	618 (53.0)
Depressive state^i^							
No	3217 (79.7)	1174 (82.3)	1051 (84.6)	123 (66.5)	1123 (81.6)	51 (72.9)	869 (74.6)
Yes	821 (20.3)	253 (17.7)	191 (15.4)	62 (33.5)	253 (18.4)	19 (27.1)	296 (25.4)

Continuous variables	Mean (SD)	Mean (SD)	Mean (SD)	Mean (SD)	Mean (SD)	Mean (SD)	Mean (SD)
Age (years)	21.4 (2.4)	21.9 (2.3)	22.0 (2.4)	21.8 (2.3)	20.5 (1.8)	21.5 (2.4)	21.7 (2.4)
Area deprivation index^j^	− 0.9 (1.7)	− 0.9 (1.7)	− 0.9 (1.7)	− 0.8 (1.5)	− 0.9 (1.7)	0.7 (1.6)	− 0.8 (1.7)

^a^Participants who replied “employed” at least once over the 3 years of follow-up

^b^Participants who replied at least once “unemployed job seeking” over the 3 years of follow-up and never “employed”

^c^Participants who only replied “student” over the 3 years of follow-up

^d^Participants who never reported their employment status over the 3 years of follow-up

^e^Alcohol Use Disorders Identification Test to evaluate alcohol use

^f^AUDIT sub-score for frequency of use by adding the scores of the first 3 items

^g^AUDIT sub-score for alcohol dependence by adding the score of items 4, 5, 6, 7, 8, 9 and 10

^h^Based on the International Standard Classification of Education

^i^Measured using the Center of Epidemiologic Studies Depression scale (CESD) and a score ≥ 19

^j^Representing spatial socioeconomic disparities

**Table 8:** Association between tobacco use and employment status over 3 years of follow-up (*n* = 1427)

Being unemployed over 3 years of follow-up									
	Model 1^a^			Model 2^b^			Model 3^c^		
	OR	95% CI		OR	95% CI		OR	95% CI	
Tobacco use									
Non-smoker	Ref	–	–	Ref	–	–	Ref	–	–
Former smoker	1.10	0.73	1.64	1.07	0.71	1.62	1.00	0.66	1.50
Current light smoker	1.20	0.85	1.70	1.18	0.83	1.67	1.06	0.74	1.52
Current moderate smoker	0.99	0.58	1.69	0.94	0.55	1.63	0.86	0.49	1.51
Current heavy smoker	2.66	0.80	8.75	2.60	0.86	7.84	1.78	0.58	5.41
Years of follow-up	0.74	0.66	0.85	0.75	0.66	0.85	0.75	0.66	0.86
Age				1.10	1.03	1.17	1.08	1.01	1.16
Gender									
Men				Ref	–	–	Ref	–	–
Women				0.93	0.72	1.21	0.85	0.66	1.10
Education level^d^				0.60	0.48	0.73	0.63	0.51	0.77
Area deprivation index^e^				0.99	0.92	1.07	1.01	0.92	1.06
Living place									
With parents				Ref	–	–	Ref	–	–
Other				0.84	0.66	1.09	0.85	0.66	1.09
Depressive state^f^									
No							Ref	–	–
Yes							2.13	1.58	2.86

^a^Univariate analysis adjusted to years of follow-up

^b^Adjusted for sociodemographic factors

^c^Adjusted for sociodemographic factors and depressive state

^d^ Based on the International Standard Classification of Education

^e^Representing spatial socioeconomic disparities

^f^Measured using the Center of Epidemiologic Studies Depression scale (CESD) and a score ≥ 19

**Table 9:** Association between cannabis use and employment status over 3 years of follow-up (*n* = 1427)

Being unemployed over 3 years of follow-up									
	Model 1^a^			Model 2^b^			Model 3^c^		
	OR	95% CI		OR	95% CI		OR	95% CI	
Cannabis consumption									
Never used	Ref	–	–	Ref	–	–	Ref	–	–
Not during the previous year	0.81	0.59	1.11	0.84	0.61	1.15	0.80	0.58	1.11
At least once during the last 12 months but less than once a month	1.10	0.75	1.59	1.16	0.80	1.69	1.08	0.74	1.59
At least once a month but less than once per week	1.04	0.66	1.66	1.12	0.69	1.80	1.06	0.66	1.71
Once per week or more	1.94	1.29	2.90	1.94	1.30	2.89	1.73	1.16	2.57
Years of follow-up	0.74	0.65	0.85	0.75	0.66	0.85	0.75	0.66	0.86
Age				1.11	1.04	1.19	1.09	1.02	1.17
Gender									
Men				Ref	–	–	Ref	–	–
Women				0.99	0.77	1.29	0.90	0.69	1.17
Education level^d^				0.60	0.49	0.74	0.63	0.52	0.78
Area deprivation index^e^				1.00	0.93	1.07	0.99	0.93	1.07
Living place									
With parents				Ref	–	–	Ref	–	–
Other				0.83	0.65	1.07	0.84	0.65	1.08
Depressive state^f^									
No							Ref	–	–
Yes							2.08	1.55	2.80

^a^Univariate analysis adjusted to years of follow-up

^b^Adjusted for sociodemographic factors

^c^Adjusted for sociodemographic factors and depressive state

^d^Based on the International Standard Classification of Education

^e^Representing spatial socioeconomic disparities

^f^Measured using the Center of Epidemiologic Studies Depression scale (CESD) and a score ≥ 19

**Table 10:** Association between alcohol use and employment status over a 3 years of follow-up (*n* = 1427)

Being unemployed over 3 years of follow-up									
	Model 1^a^			Model 2^b^			Model 3^c^		
	OR	95% CI		OR	95% CI		OR	95% CI	
AUDIT^d^									
Low risk	Ref	–	–	Ref	–	–	Ref	–	–
No use	1.85	1.12	3.07	1.57	0.93	2.64	1.50	0.88	2.53
At risk	1.31	1.01	1.72	1.35	1.02	1.78	1.31	0.99	1.72
Years of follow-up	0.74	0.65	0.84	0.75	0.65	0.85	0.75	0.66	0.86
Age				1.10	1.02	1.17	1.08	1.01	1.15
Gender									
Men				Ref	–	–	Ref	–	–
Women				0.99	0.76	1.29	0.90	0.69	1.17
Education level^e^				0.60	0.49	0.74	0.64	0.52	0.78
Area deprivation index^f^				0.99	0.92	1.07	0.99	0.92	1.06
Living place									
With parents				Ref	–	–	Ref	–	–
Other				0.84	0.65	1.08	0.84	0.65	1.08
Depressive state^g^									
No							Ref	–	–
Yes							2.12	1.58	2.84

^a^Univariate analysis adjusted to years of follow-up

^b^Adjusted for sociodemographic factors

^c^Adjusted for sociodemographic factors and depressive state

^d^Alcohol Use Disorders Identification Test to evaluate alcohol use

^e^Based on the International Standard Classification of Education

^f^Representing spatial socioeconomic disparities

^g^Measured using the Center of Epidemiologic Studies Depression scale (CESD) and a score ≥ 19

**Table 11:** Association between alcohol use and employment status over a 3 years of follow-up by differentiating between frequency of use and dependence (*n* = 1427)

Being unemployed over 3 years of follow-up									
	Model 1^a^			Model 2^b^			Model 3^c^		
	OR	95% CI		OR	95% CI		OR	95% CI	
Alcohol frequency of use^d^									
[0–2]	Ref	–	–	Ref	–	–	Ref	–	–
[3–5]	0.82	0.59	1.12	0.87	0.63	1.21	0.84	0.60	1.17
[6 +]	0.74	0.52	1.05	0.84	0.98	1.20	0.82	0.57	1.16
Alcohol dependence^e^									
[0]	Ref	–	–	Ref	–	–	Ref	–	–
[1–2]	1.24	0.91	1.69	1.21	0.89	1.64	1.18	0.86	1.60
[3 +]	1.80	1.27	2.54	1.74	1.23	2.46	1.65	1.16	2.34
Years of follow-up	0.75	0.66	0.85	0.75	0.66	0.86	0.76	0.66	0.87
Age				1.10	1.03	1.17	1.08	1.01	1.15
Gender									
Men				Ref	–	–	Ref	–	–
Women				0.96	0.73	1.25	0.87	0.66	1.13
Education level^f^				0.60	0.49	0.74	0.63	0.52	0.78
Area deprivation index^g^				0.99	0.92	1.07	0.99	0.92	1.07
Living place									
With parents				Ref	–	–	Ref	–	–
Other				0.85	0.66	1.09	0.85	0.66	1.09
Depressive state^h^									
No							Ref	–	–
Yes							2.11	1.58	2.83

^a^Univariate analysis adjusted to years of follow-up

^b^Adjusted for sociodemographic factors

^c^Adjusted for sociodemographic factors and depressive state

^d^AUDIT sub-score for frequency of use by adding the scores of the first 3 items

^e^AUDIT sub-score for alcohol dependence by adding the score of items 4, 5, 6, 7, 8, 9 and 10

^f^Based on the International Standard Classification of Education

^g^Representing spatial socioeconomic disparities

^h^Measured using the Center of Epidemiologic Studies Depression scale (CESD) and a score ≥ 19

**Table 12:** Association between alcohol dependence and cannabis use once per week or more with employment status over 3-year follow-up (*n* = 1427)

Being unemployed over 3 years of follow-up									
	Model 1^a^			Model 2^b^			Model 3^c^		
	OR	95% CI		OR	95% CI		OR	95% CI	
Substance use									
Alcohol dependence^d^ score < 3 and cannabis use less than once per week	Ref	–	–	Ref	–	–	Ref	–	–
Alcohol dependence score ≥ 3 or cannabis use once per week or more	1.47	1.09	1.98	1.48	1.10	2.01	1.43	1.05	1.93
Alcohol dependence score ≥ 3 and cannabis use once per week or more	2.74	1.56	4.80	2.75	1.60	4.72	2.44	1.44	4.12
Years of follow-up				0.76	0.66	0.86	0.76	0.67	0.87
Age				1.10	1.03	1.18	1.09	1.02	1.16
Gender									
Men				Ref	–	–	Ref	–	–
Women				1.01	0.78	1.31	0.91	0.71	1.19
Education level^e^				0.60	0.49	0.74	0.63	0.51	0.78
Area deprivation index^f^				1.01	0.93	1.08	1.01	0.93	1.08
Living place									
With parents				Ref	–	–	Ref	–	–
Other				0.83	0.65	1.07	0.84	0.65	1.08
Depressive state^g^									
No							Ref	–	–
Yes							2.05	1.53	2.75

^a^Univariate analysis adjusted to years of follow-up

^b^Adjusted for sociodemographic factors

^c^Adjusted for sociodemographic factors and depressive state

^d^AUDIT sub-score for alcohol dependence by adding the score of items 4, 5, 6, 7, 8, 9 and 10

^e^Based on the International Standard Classification of Education

^f^Representing spatial socioeconomic disparities

^g^Measured using the Center of Epidemiologic Studies Depression scale (CESD) and a score ≥ 19

## Discussion

We aimed to examine prospectively the associations between tobacco, cannabis and, alcohol and subsequent employment status in a large sample of students or young people in training at inclusion. Over a 3-year follow-up period, at least weekly cannabis use and alcohol dependence were negatively associated with employment, even after adjusting for sociodemographic characteristics and depressive state. Our results suggested that alcohol dependence was associated with employment to a greater extent than the frequency of alcohol use. No significant associations were observed for tobacco use.

To the best of our knowledge, the present study is the first to examine prospectively the role of tobacco, cannabis, and alcohol use on the likelihood of accessing employment. In addition, our analyses take into consideration sociodemographic factors and depressive state measured with a validated assessment tool. Nevertheless, this study has some limitations. First, even if the participants from the CONSTANCES cohort were randomly recruited, participants who decided to be involved in a cohort for health and research purposes differ from the general population. They tend to be healthier, consume less tobacco, cannabis or alcohol, have higher education level and socioeconomic status [39]. Thus, while selecting the subsample of young participants, these characteristics might contribute to the higher prevalence of women compared to men. Moreover, our sample was composed of 95% French native participants thus, limiting our conclusion regarding the role of substance use on employment status in foreign young people. Second, 28.8% of the participants did not report their employment status during the follow-ups. However, young participants have a low response rate at follow-up, which has been already observed in other cohorts [40]. Therefore, even in the case of missing data at follow-up, this would only have led to an underestimation of the associations between substance use and employment. If we consider those with three follow-up points, there are 26% with missing data. However, there was little difference between respondents and participants with missing data in terms of education background, reducing the bias from socioeconomic inequalities. Moreover, even though loss to follow-up is inevitable in cohort studies, non-response due to attrition unlikely causes bias in the examined associations as shown in several studies, more specifically on alcohol and smoking intake [41–44]. Third, although our study was prospective, its observational nature prevents causal conclusions as other unmeasured confounding factors may play a role in the associations between substance use and employment (e.g., adverse childhood events, personality traits). Although our study was prospective, its observational nature prevents causal conclusions as other unmeasured confounding factors may play a role in the associations between substance use and employment (e.g., adverse childhood events, personality traits). However, education and depression, two variables known to be strongly associated with both substance use and employment, had strengths of associations of 1.47 and 2.13, respectively [19]. Thus, according to the calculated e-values (i.e., 2.85 for cannabis use once per week or more and 2.68 for alcohol dependence), unmeasured confounding factors should have a greater effect size than education or depression to fully explain the reported associations, which seems rather unlikely. Third, employment status did not include unpaid activities (e.g., unpaid internship, undeclared job, and homemaker) which we are not able to control for in this study.

Participants who reported using cannabis at least weekly had decreased odds of being employed at follow-up. Every day or almost every day cannabis use is usually referred to as heavy use [45] and is known to be associated with harmful health and psychosocial adverse effects [46]. The acute adverse effects of cannabis include anxiety, panic reactions and psychotic symptoms [45]. In addition, regular use initiated during adolescence was reported to impair cognitive performances [47], especially attention and concentration, information processing, motor performance, and tasks related to inhibition [47, 48]. All these adverse effects may impact one’s job searching, recruitment after a job interview and at the end, the access to employment. Notably, after adjusting for depressive state, the effect size of the association between cannabis use and employment decreased, highlighting the important role of depression on this association whatever the underlined mechanisms (i.e., confounding and/or mediating effect). Therefore, our results also recall the importance of treating depression in young adults in order to increase their chances in accessing employment.

At-risk alcohol use was associated with decreased odds of employment before adjustment for depressive state. Moreover, our findings suggest that symptoms of dependency could be more associated with difficulties of accessing first employment than with the frequency of use. It is likely that symptoms of alcohol dependency could have more severe consequences on employment than alcohol use per se because people with alcohol dependence may experience difficulty to decrease their consumption before critical moments such as job interview or first days in employment and thus suffer from disabling symptoms (e.g., disinhibition, blackouts, and withdrawal symptoms including irritability) [49]. In line with these hypotheses, Bamberger et al. found that the alcohol frequency of use was not associated with access to employment among university graduating seniors but only the frequency of heavy episodic drinking in a survey that did not assess specifically dependence symptoms [23].

Although not statistically significant, we found higher odds of unemployment in heavy smokers compared to non-smokers. Since very few participants were heavy smokers (*n* = 11), non-significance may be due to a lack of statistical power. Thus, this finding should not be interpreted as a lack of association between smoking and unemployment. Future studies should try to address this issue, for instance by oversampling heavy smokers.

The use of both cannabis and alcohol was associated with a higher odd of unemployment compared to the use of a single substance but these odds were not significantly different. This finding suggests that cannabis use once per week and more or alcohol dependence are sufficient to experience an increased likelihood of unemployment. Sensitivity analyses examined whether our results could be explained by a small group of subjects who were particularly at risk for both substance use and employment. However, the associations persisted after excluding participants with psychiatric history as well as after excluding participants who initiated alcohol use before the age of 13. These findings suggest that the role of substance use on youth employment concern potentially all the youth and not only certain subgroups particularly at risk. However, since associations between cannabis use and employment prevailed in youth from lower education level, preventive strategies should tackle first these individuals.

Public health campaigns are needed among youth to tackle the detrimental role of even weekly cannabis use and at-risk alcohol use by highlighting the potential substantial benefits of decreasing their substance use, not only for health reasons, but also to enhance their ability to get a job. Such information could motivate young people to seek in proper help. This could be particularly relevant since unemployment is associated with detrimental mental health [50] and, including substance use, leading to a vicious circle. Prevention strategies should be implemented in training and job search organizations that are targeted by youth, such as a standardized tool for screening substance use before it gets aggravated by prolonged unemployment. Further studies should be conducted to assess the benefits of such preventive strategies in substance use on subsequent access to employment in youth, also tackling other substances such as cocaine. In addition, since the role of substance use on employment might differ according to financial support from government or parents for some students, this information should be considered in future studies.

## Supplementary Information

Below is the link to the electronic supplementary material.

Supplementary file1 (DOCX 91 KB)

## Data Availability

Personal health data underlying the findings of our study are not publicly available due to legal reasons related to data privacy protection. CONSTANCES has a data sharing policy but before data transfer a legal authorization has to be obtained from the CNIL (Commission nationale de l’informatique et des libertés), the French data privacy authority. The CONSTANCES email address is contact@constances.fr.

## Code availability

The code is available from the first author (REH), upon reasonable request.
