# Supplementary material for: The association between substance use and subsequent employment among students: prospective findings from the CONSTANCES cohort
Source: Soc Psychiatry Psychiatr Epidemiol. 2022 Sep 2;58(2):249–66. doi: 10.1007/s00127-022-02357-0 (PMC9437401; doi:10.1007/s00127-022-02357-0)
Supplement: Supplementary file 1 — Supplementary file1 (DOCX 91 KB) [file 127_2022_2357_MOESM1_ESM.docx]

Supplemental tables

Supplemental Table 1. Characteristics of the 1,427 (562 employed, 173 unemployed at all forms, 533 students and 159 with missing data at one-year follow-up) participants included according to the employment status at each year of follow-up.

|  | **Employed at 1 year** | | | **Employed at 2 years** | | | | **Employed at 3 years** | | | |
| --- | --- | --- | --- | --- | --- | --- | --- | --- | --- | --- | --- |
|  | Yes  562 (44.3) | No  173 (13.7) | Student  533 (42.0) | Yes  671 (58.8) | No  164 (14.4) | Student  306 (26.8) | Yes  742 (78.8) | | No  128 (13.6) | Student  72 (7.6) |  |
| **CATEGORICAL VARIABLES** | N (%) | N (%) | N (%) | N (%) | N (%) | N (%) | N (%) | | N (%) | N (%) |  |
| **Tobacco use** |  |  |  |  |  |  |  | |  |  |  |
| Non-smoker | 371 (66.0) | 110 (63.6) | 376 (70.5) | 470 (70.0) | 99 (60.4) | 218 (71.1) | 517 (69.7) | | 88 (68.7) | 49 (68.1) |  |
| Former smoker | 52 (9.3) | 25 (14.5) | 48 (9.0) | 67 (10.0) | 14 (8.5) | 28 (9.2) | 69 (9.3) | | 10 (7.8) | 6 (8.3) |  |
| Current light smoker | 86 (15.3) | 26 (15.0) | 71 (13.3) | 96 (14.3) | 33 (20.1) | 37 (12.1) | 99 (13.3) | | 23 (18.0) | 11 (15.3) |  |
| Current moderate Smoker | 50 (8.9) | 8 (4.6) | 37 (7.0) | 36 (5.4) | 16 (9.8) | 21 (6.9) | 51 (6.9) | | 7 (5.5) | 6 (8.3) |  |
| Current heavy smoker | 3 (0.53) | 4 (2.3) | 1 (0.2) | 2 (0.3) | 2 (1.2) | 2 (0.7) | 6 (0.8) | | 0 (0.0) | 0 (0.0) |  |
| **Cannabis Consumption** |  |  |  |  |  |  |  | |  |  |  |
| Never used | 259 (46.1) | 74 (42.8) | 283 (53.1) | 331 (49.3) | 70 (42.7) | 160 (52.3) | 376 (50.7) | | 64 (50.0) | 30 (41.7) |  |
| Not during the previous year | 146 (26.0) | 41 (23.7) | 97 (18.2) | 165 (24.6) | 30 (18.3) | 54 (17.6) | 158 (21.3) | | 22 (17.2) | 11 (15.3) |  |
| At least once during the last 12 months but less than once a month | 68 (12.1) | 19 (11.0) | 75 (14.1) | 79 (11.8) | 22 (13.4) | 53 (17.3) | 102 (13.8) | | 22 (17.2) | 16 (22.2) |  |
| At least once a month but less than once per week | 37 (6.6) | 16 (9.2) | 46 (8.6) | 60 (8.9) | 12 (7.3) | 15 (4.9) | 53 (7.1) | | 7 (5.5) | 5 (6.9) |  |
| Once per week or more | 52 (9.2) | 23 (13.3) | 32 (6.0) | 36 (5.4) | 30 (18.3) | 24 (7.8) | 53 (7.1) | | 13 (10.2) | 10 (13.9) |  |
| **AUDIT Score^a^** |  |  |  |  |  |  |  | |  |  |  |
| 0 | 24 (4.3) | 15 (8.7) | 23 (4.3) | 30 (4.5) | 12 (7.3) | 13 (4.2) | 36 (4.8) | | 8 (6.3) | 2 (2.8) |  |
| 1-7 | 396 (70.4) | 11 (64.2) | 375 (70.4) | 491 (73.2) | 94 (57.3) | 222 (72.5) | 514 (69.3) | | 87 (68.0) | 49 (68.1) |  |
| >7 | 142 (25.3) | 47 (27.1) | 135 (25.3) | 150 (22.3) | 58 (35.4) | 71 (23.2) | 192 (25.9) | | 33 (25.7) | 21 (29.1) |  |
| **Alcohol Frequency of Use^b^** |  |  |  |  |  |  |  | |  |  |  |
| [0-2] | 180 (32.0) | 59 (34.1) | 179 (33.6) | 224 (33.4) | 53 (32.3) | 106 (34.6) | 233 (31.4) | | 42 (32.8) | 23 (31.9) |  |
| [3-5] | 213 (37.9) | 69 (39.9) | 196 (36.8) | 272 (40.5) | 55 (33.5) | 112 (36.6) | 288 (38.8) | | 52 (40.6) | 28 (38.9) |  |
| [6+] | 169 (30.1) | 45 (26.0) | 158 (29.6) | 175 (26.1) | 56 (34.2) | 88 (28.8) | 221 (29.8) | | 34 (26.6) | 21 (29.2) |  |
| **Alcohol Dependence^c^** |  |  |  |  |  |  |  | |  |  |  |
| 0 | 276 (49.1) | 75 (43.3) | 278 (52.2) | 331 (49.3) | 66 (40.2) | 163 (53.3) | 384 (51.8) | | 61 (47.7) | 34 (47.2) |  |
| [1-2] | 172 (30.6) | 55 (31.8) | 160 (30.0) | 215 (32.1) | 49 (29.9) | 92 (30.1) | 222 (29.9) | | 36 (28.1) | 22 (30.6) |  |
| [3+] | 114 (20.3) | 43 (24.9) | 95 (17.8) | 125 (18.6) | 49 (29.9) | 49 (16.0) | 136 (18.3) | | 31 (24.2) | 16 (22.2) |  |
| **Gender** |  |  |  |  |  |  |  | |  |  |  |
| Men | 157 (27.9) | 56 (32.4) | 173 (32.5) | 211 (31.4) | 54 (32.9) | 91 (29.7) | 231 (31.1) | | 40 (31.3) | 20 (27.8) |  |
| Women | 405 (72.1) | 117 (67.6) | 360 (67.5) | 460 (68.5) | 110 (67.1) | 215 (70.3) | 511 (68.9) | | 88 (68.7) | 52 (72.2) |  |
| **Education^d^** |  |  |  |  |  |  |  | |  |  |  |
| Early childhood or primary | 1 (0.2) | 2 (1.2) | 1 (0.2) | 1 (0.1) | 1 (0.6) | 2 (0.7) | 2 (0.3) | | 0(0.0) | 0 (0.0) |  |
| Lower secondary | 8 (1.4) | 10 (5.8) | 8 (1.5) | 7 (1.1) | 11 (6.7) | 6 (2.0) | 6 (0.8) | | 4 (3.1) | 2 (2.8) |  |
| Upper secondary or post-secondary non-tertiary | 228 (40.6) | 74 (42.8) | 267 (50.1) | 291 (43.4) | 77 (47.0) | 151 (49.3) | 306 (41.2) | | 60 (46.9) | 45 (62.5) |  |
| Short-cycle tertiary or bachelor’s | 202 (35.9) | 52 (30.0) | 222 (41.6) | 253 (37.7) | 62 (37.8) | 123 (40.2) | 316 (42.6) | | 52 (40.6) | 17 (23.6) |  |
| Master’s or doctoral | 123 (21.9) | 35 (20.2) | 35 (6.6) | 119 (17.7) | 13 (7.9) | 24 (7.8) | 112 (15.1) | | 12 (9.4) | 8 (11.1) |  |
| **Living Place** |  |  |  |  |  |  |  | |  |  |  |
| With parents | 274 (48.8) | 92 (53.2) | 285 (53.5) | 325 (48.4) | 87 (53.0) | 182 (59.5) | 356 (48.0) | | 72 (56.2) | 49 (68.1) |  |
| Others | 288 (51.2) | 81 (46.8) | 248 (46.5) | 246 (36.6) | 77 (47.0) | 124 (40.5) | 386 (52.0) | | 56 (43.8) | 23 (31.9) |  |
| **Parents’ Highest Occupational Grade** |  |  |  |  |  |  |  | |  |  |  |
| Home or other | 3 (0.6) | 1(0.6) | 4 (0.8) | 3 (0.5) | 4 (2.4) | 1 (0.3) | 4 (0.5) | | 2 (1.6) | 0 (0.0) |  |
| Blue-collar worker, craftsman, farmer or employee | 216 (38.4) | 62 (35.8) | 169 (31.7) | 233 (34.7) | 68 (41.5) | 102 (33.3) | 245 (33.0) | | 42 (32.8) | 27 (37.5) |  |
| Intermediate worker | 144 (25.6) | 45 (26.0) | 128 (24.0) | 162 (24.1) | 36 (22.0) | 70 (22.9) | 184 (24.7) | | 31 (24.2) | 16 (22.2) |  |
| Executive, higher intellectual profession | 199 (35.4) | 65 (37.6) | 232 (43.5) | 273 (40.7) | 56 (34.1) | 133 (43.5) | 309 (41.6) | | 53 (41.4) | 29 (40.3) |  |
| **Depressive State^e^** |  |  |  |  |  |  |  | |  |  |  |
| No | 472 (84.0) | 126 (72.8) | 450 (84.4) | 564 (84.1) | 111 (67.7) | 260 (85.0) | 641 (86.4) | | 94 (73.4) | 51 (70.8) |  |
| Yes | 56 (16.0) | 47 (27.2) | 83 (15.6) | 107 (15.9) | 53 (32.3) | 46 (15.0) | 101 (13.6) | | 34 (26.6) | 21 (29.2) |  |
| **CONTINUOUS VARIABLES** | **Mean (SD)** | **Mean (SD)** | **Mean (SD)** | **Mean (SD)** | **Mean (SD)** | **Mean (SD)** | **Mean (SD)** | | **Mean (SD)** | **Mean (SD)** |  |
| **Age** (years) | 22.4 (2.5) | 22.6 (2.6) | 21.4 (1.9) | 22.2 (2.3) | 21.8(2.3) | 21.3 (1.9) | 22.1 (2.2) | | 21.9 (2.2) | 20.8 (2.2) |  |
| **Area Deprivation Index^f^** | -0.9 (1.7) | -0.8 (1.7) | -1.0 (1.6) | -0.9 (1.7) | -0.9 (1.6) | -0.9 (1.6) | -1.1 (1.7) | | -0.9 (1.6) | -0.8 (1.8) |  |
| \| **^a^** Alcohol Use Disorders Identification Test to evaluate alcohol use  ^b^ AUDIT sub-score for frequency of use by adding the scores of the first 3 items  ^c^ AUDIT sub-score for alcohol dependence by adding the score of items 4, 5, 6, 7, 8, 9 and 10  ^d^ Based on the International Standard Classification of Education  ^e^ Measured using the Center of Epidemiologic Studies Depression scale (CESD) and a score ≥19  ^f^ Representing spatial socioeconomic disparities \| \| --- \| | | | | | | | | | |  |  |

Table 2. Characteristics of participants with missing data according to their number of follow-ups (N= 4,038)..

|  | **Participants with one-year follow-up (N=420)** | | **Participants with two-year follow-up (N=547)** | | | **Participants with three-year follow-up (N=3,071)** | | | |
| --- | --- | --- | --- | --- | --- | --- | --- | --- | --- |
| **CATEGORICAL VARIABLES** | **Not missing**  **(N=234)** | **Missing**  **(N=186)** | **Not missing**  **(N=291)** | **One missing follow-up**  **(N=96)** | **Two missing follow-ups**  **(N=160)** | **Not missing**  **(N=1,259)** | **One missing follow-up**  **(N=518)** | **Two missing follow-ups**  **(N=475)** | **Three missing follow-ups**  **(N=819)** |
| **Tobacco use** |  |  |  |  |  |  |  |  |  |
| Non-smoker | 175 (74.8) | 113 (60.7) | 212 (72.8) | 53 (55.2) | 89 (55.6) | 923 (73.3) | 336 (64.9) | 297 (62.5) | 496 (60.6) |
| Former smoker | 24 (10.3) | 16 (8.6) | 29 (10.0) | 11 (11.5) | 14 (8.8) | 105 (8.3) | 54 (10.4) | 53 (11.2) | 83 (10.1) |
| Current light smoker | 27 (11.5) | 42 (22.6) | 36 (12.4) | 20 (20.8) | 37 (23.1) | 164 (13.0) | 85 (16.4) | 85 (17.9) | 148 (18.1) |
| Current moderate Smoker | 7 (3.0) | 13 (7.0) | 12 (4.1) | 11 (11.5) | 17 (10.6) | 62 (5.0) | 38 (7.3) | 33 (6.9) | 82 (10.0) |
| Current heavy smoker | 1 (0.4) | 2 (1.1) | 2 (0.7) | 1 (1.0) | 3 (1.9) | 5 (0.4) | 5 (1.0) | 7 (1.5) | 10 (1.2) |
| **Cannabis Consumption** |  |  |  |  |  |  |  |  |  |
| Never used | 120 (51.3) | 80 (43.0) | 158 (54.3) | 32 (33.3) | 67 (41.9) | 632 (50.2) | 247 (47.7) | 209 (44.0) | 344 (42.0) |
| Not during the previous year | 38 (16.2) | 37 (19.9) | 65 (22.3) | 25 (26.1) | 31 (19.4) | 251 (19.9) | 86 (16.6) | 104 (21.9) | 164 (20.0) |
| At least once during the last 12 months but less than once a month | 41 (17.5) | 29 (15.6) | 31 (10.7) | 13 (13.5) | 21 (13.1) | 202 (16.0) | 76 (14.7) | 56 (11.8) | 165 (12.8) |
| At least once a month but less than once per week | 20 (8.5) | 17 (9.1) | 21 (7.2) | 10 (10.4) | 14 (8.8) | 86 (6.8) | 48 (9.3) | 42 (8.8) | 74 (9.0) |
| Once per week or more | 15 (6.4) | 23 (12.4) | 16 (5.5) | 16 (16.7) | 27 (16.9) | 88 (7.0) | 61 (11.8) | 64 (13.5) | 132 (16.1) |
| **AUDIT Score^a^** |  |  |  |  |  |  |  |  |  |
| 0 | 12 (5.1) | 17 (9.1) | 16 (5.5) | 4 (4.2) | 19 (11.9) | 60 (4.8) | 26 (5.0) | 32 (6.7) | 87 (10.6) |
| 1-7 | 156 (66.7) | 111 (59.7) | 203 (69.8) | 64 (66.7) | 94 (58.8) | 887 (70.4) | 345 (66.6) | 291 (61.3) | 473 (57.8) |
| >7 | 66 (28.2) | 58 (31.2) | 72 (24.7) | 28 (29.1) | 47 (29.4) | 312 (24.8) | 147 (28.4) | 152 (32.0) | 259 (31.6) |
| **Alcohol Frequency of Use^b^** |  |  |  |  |  |  |  |  |  |
| [0-2] | 75 (32.1) | 59 (31.7) | 115 (39.5) | 29 (30.2) | 45 (28.1) | 423 (33.6) | 153 (29.5) | 158 (33.3) | 273 (33.3) |
| [3-5] | 95 (40.6) | 73 (39.3) | 97 (33.3) | 29 (30.2) | 64 (40.0) | 488 (38.8) | 190 (36.7) | 168 (35.4) | 285 (34.8) |
| [6+] | 64 (27.3) | 54 (29.0) | 79 (27.2) | 38 (39.6) | 61 (31.9) | 348 (37.6) | 175 (33.8) | 149 (31.4) | 261 (31.9) |
| **Alcohol Dependence^c^** |  |  |  |  |  |  |  |  |  |
| 0 | 112 (47.9) | 86 (46.2) | 144 (49.5) | 41 (42.7) | 71 (44.4) | 637 (50.6) | 246 (47.5) | 223 (47.0) | 377 (46.0) |
| [1-2] | 78 (33.3) | 50 (26.9) | 84 (28.9) | 35 (36.5) | 51 (31.9) | 377 (29.9) | 158 (30.5) | 125 (26.3) | 226 (27.6) |
| [3+] | 44 (18.8) | 50 (26.9) | 63 (21.6) | 20 (20.8) | 37 (23.1) | 245 (19.5) | 114 (22.0) | 127 (26.7) | 216 (26.4) |
| **Gender** |  |  |  |  |  |  |  |  |  |
| Men | 77 (32.9) | 91 (48.9) | 108 (37.1) | 52 (54.2) | 76 (47.5) | 401 (31.9) | 186 (35.9) | 176 (37.1) | 314 (38.4) |
| Women | 157 (67.1) | 95 (51.1) | 183 (62.9) | 44 (45.8) | 84 (52.5) | 858 (68.1) | 332 (64.1) | 299 (62.9) | 505 (61.7) |
| **Education^d^** |  |  |  |  |  |  |  |  |  |
| Early childhood or primary | 2 (0.9) | 0 (0.0) | 3 (1.0) | 1 (1.0) | 3 (1.9) | 2 (0.2) | 4 (0.8) | 2 (0.4) | 4 (0.5) |
| Lower secondary | 11 (4.7) | 11 (5.9) | 13 (4.5) | 8 (8.3) | 8 (5.0) | 20 (1.6) | 14 (2.7) | 16 (3.3) | 29 (3.5) |
| Upper secondary or post-secondary non-tertiary | 152 (65.0) | 129 (69.4) | 210 (72.2) | 62 (64.6) | 100 (62.5) | 684 (54.3) | 258 (49.8) | 253 (53.3) | 388 (47.4) |
| Short-cycle tertiary or bachelor’s | 49 (20.9) | 34 (18.3) | 40 (13.7) | 19 (19.8) | 26 (16.3) | 437 (34.7) | 186 (35.9) | 159 (33.5) | 297 (36.3) |
| Master’s or doctoral | 20 (8.5) | 12 (6.4) | 25 (8.6) | 6 (6.3) | 23 (14.3) | 117 (9.3) | 56 (10.8) | 45 (9.5) | 101 (12.3) |
| **Living Place** |  |  |  |  |  |  |  |  |  |
| With parents | 131 (56.0) | 114 (61.3) | 181 (62.2) | 61 (63.6) | 88 (55.0) | 698 (55.4) | 266 (51.4) | 232 (48.8) | 345 (42.1) |
| Others | 103 (44.0) | 72 (38.7) | 110 (37.8) | 35 (36.4) | 72 (45.0) | 56 (44.6) | 252 (48.6) | 243 (51.2) | 474 (57.9) |
| **Depressive State^e^** |  |  |  |  |  |  |  |  |  |
| No | 184 (78.6) | 146 (78.5) | 238 (81.8) | 70 (72.9) | 119 (74.4) | 1,050 (83.4) | 423 (81.7) | 323 (80.6) | 605 (73.9) |
| Yes | 50 (21.4) | 40 (21.5) | 53 (18.2) | 26 (27.1) | 41 (25.6) | 209 (16.6) | 95 (18.3) | 92 (19.4) | 214 (26.1) |
| **CONTINUOUS VARIABLES** | **Mean (SD)** | **Mean (SD)** | **Mean (SD)** | **Mean (SD)** | **Mean (SD)** | **Mean (SD)** | **Mean (SD)** | **Mean (SD)** | **Mean (SD)** |
| **Age** (years) | 20.7 (2.1) | 21.1 (2.2) | 20.7 (2.4) | 20.6 (2.1) | 21.3 (2.5) | 21.3 (2.1) | 21.5 (2.2) | 21.6 (2.3) | 21.9 (2.4) |
| **Area Deprivation Index^f^** | -0.6 (1.7) | -0.7 (1.6) | -0.6 (1.6) | -0.8 (1.5) | -0.3 (1.8) | -1.1 (1.7) | -1.1 (1.7) | -0.8 (1.6) | -0.9 (1.7) |
| \| **^a^** Alcohol Use Disorders Identification Test to evaluate alcohol use  ^b^ AUDIT sub-score for frequency of use by adding the scores of the first 3 items  ^c^ AUDIT sub-score for alcohol dependence by adding the score of items 4, 5, 6, 7, 8, 9 and 10  ^d^ Based on the International Standard Classification of Education  ^e^ Measured using the Center of Epidemiologic Studies Depression scale (CESD) and a score ≥19  ^f^ Representing spatial socioeconomic disparities \| \| --- \| | | | | | | | | | |

Supplemental Table 3. Association between cannabis use and employment status over a three-year of follow-up stratified on education (n=1,427).

| **No access to employment over three -years of follow-up** | | | | | | | | | | |
| --- | --- | --- | --- | --- | --- | --- | --- | --- | --- | --- |
|  |  | **Model 1^a^** | | | **Model 2^b^** | | | **Model 3^c^** | | |
|  |  | **OR** | **95% CI** | | **OR** | **95% CI** | | **OR** | **95% CI** | |
| **Less than a post-secondary non-tertiary**  **N=686** | **Cannabis Consumption** |  |  |  |  |  |  |  |  |  |
|  | Never used | Ref | - | - | Ref | - | - | Ref | - | - |
|  | Not during the previous year | **0.61** | **0.36** | **1.01** | **0.56** | **0.33** | **0.93** | **0.53** | **0.31** | **0.92** |
|  | At least once during the last 12 months but less than once a month | 0.73 | 0.41 | 1.29 | 0.76 | 0.42 | 1.34 | 0.68 | 0.38 | 1.21 |
|  | At least once a month but less than once per week | 0.71 | 0.34 | 1.45 | 0.75 | 0.37 | 1.54 | 0.69 | 0.34 | 1.43 |
|  | Once per week or more | **2.20** | **1.32** | **3.63** | **2.35** | **1.41** | **3.94** | **2.06** | **1.25** | **3.41** |
| **Post-secondary non-tertiary or more**  **N=741** | **Cannabis Consumption** |  |  |  |  |  |  |  |  |  |
|  | Never used | Ref | - | - | Ref | - | - | Ref | - | - |
|  | Not during the previous year | 1.13 | 0.74 | 1.71 | 1.14 | 0.74 | 1.71 | 1.07 | 0.69 | 1.64 |
|  | At least once during the last 12 months but less than once a month | **1.66** | **1.01** | **2.74** | **1.65** | **1.01** | **2.73** | 1.58 | 0.94 | 2.66 |
|  | At least once a month but less than once per week | 1.59 | 0.86 | 2.93 | 1.61 | 0.86 | 3.02 | 1.54 | 0.80 | 2.96 |
|  | Once per week or more | 1.69 | 0.88 | 3.27 | 1.70 | 0.88 | 3.30 | 1.50 | 0.77 | 2.91 |
| ^a^ Univariate analysis adjusted to years of follow-up.  ^b^ Adjusted for sociodemographic factors  ^c^ Adjusted for sociodemographic factors and depressive state | | | | | | | | | | |

Supplemental Table 4. Association between tobacco use and access to employment over a three-year of follow-up after adjusting for parents’ highest occupational grade (n=1,427).

| **Being unemployed over three -years of follow-up** | | | |
| --- | --- | --- | --- |
|  | **OR** | **95% CI** | |
| **Tobacco Use** |  |  |  |
| Non-smoker | Ref | - | - |
| Former smoker | 1.01 | 0.66 | 1.50 |
| Current light smoker | 1.07 | 0.75 | 1.54 |
| Current moderate smoker | 0.87 | 0.50 | 1.53 |
| Current heavy smoker | 1.79 | 0.59 | 5.45 |
| **Years of Follow-up** | **0.75** | **0.66** | **0.86** |
| **Age** | 1.08 | 1.01 | 1.16 |
| **Gender** |  |  |  |
| Men | Ref | - | - |
| Women | **0.85** | **0.66** | **1.11** |
| **Education Level^a^** | **0.63** | **0.51** | **0.77** |
| **Area Deprivation Index^b^** | 0.99 | 0.92 | 1.07 |
| **Living Place** |  |  |  |
| With parents | Ref | - | - |
| Other | 0.84 | 0.65 | 1.09 |
| **Parents’ Highest Occupational Grade** |  |  |  |
| Home or other | Ref | - | - |
| Blue-collar worker, craftsman, farmer or employee | 0.52 | 0.14 | 1.91 |
| Intermediate worker | 0.48 | 0.12 | 1.85 |
| Executive, higher intellectual profession | 0.55 | 0.14 | 2.11 |
| **Depressive State^c^** |  |  |  |
| No | Ref | - | - |
| Yes | **2.11** | **1.56** | **2.84** |
| ^a^ Based on the International Standard Classification of Education  ^b^ Representing spatial socioeconomic disparities  ^c^ Measured using the Center of Epidemiologic Studies Depression scale (CESD) and a score ≥19 | | | |

Supplemental Table 5. Association between cannabis use and access to employment over a three-year of follow-up after adjusting for parents’ highest occupational grade (n=1,427).

| **Being unemployed over three -years of follow-up** | | | |
| --- | --- | --- | --- |
|  | **OR** | **95% CI** | |
| **Cannabis Consumption** |  |  |  |
| Never used | Ref | - | - |
| Not during the previous year | 0.80 | 0.58 | 1.10 |
| At least once during the last 12 months but less than once a month | 1.07 | 0.73 | 1.59 |
| At least once a month but less than once per week | 1.03 | 0.63 | 1.69 |
| Once per week or more | **1.72** | **1.15** | **2.55** |
| **Years of Follow-up** | **0.75** | **0.66** | **0.86** |
| **Age** | 1.09 | 1.02 | 1.17 |
| **Gender** |  |  |  |
| Men | Ref | - | - |
| Women | 0.90 | 0.69 | 1.17 |
| **Education Level^a^** | **0.63** | **0.52** | **0.78** |
| **Area Deprivation Index^b^** | 1.01 | 0.93 | 1.08 |
| **Living Place** |  |  |  |
| With parents | Ref | - | - |
| Other | 0.83 | 0.65 | 1.08 |
| **Parents’ Highest Occupational Grade** |  |  |  |
| Home or other | Ref | - | - |
| Blue-collar worker, craftsman, farmer or employee | 0.57 | 0.15 | 2.12 |
| Intermediate worker | 0.53 | 0.13 | 2.04 |
| Executive, higher intellectual profession | 0.61 | 0.16 | 2.35 |
| **Depressive State^c^** |  |  |  |
| No | Ref | - | - |
| Yes | **2.08** | **1.54** | **2.79** |
| ^a^ Based on the International Standard Classification of Education  ^b^ Representing spatial socioeconomic disparities  ^c^ Measured using the Center of Epidemiologic Studies Depression scale (CESD) and a score ≥19 | | | |

Supplemental Table 6. Association between alcohol use and access to employment over a three-year of follow-up after adjusting for parents’ highest occupational grade (n=1.427).

| **Being unemployed over three -years of follow-up** | | | |
| --- | --- | --- | --- |
|  | **OR** | **95% CI** | |
| **AUDIT^a^** |  |  |  |
| Low risk | Ref | - | - |
| No use | 1.48 | 0.87 | 2.50 |
| At risk | 1.31 | 0.99 | 1.73 |
| **Years of Follow-up** | **0.75** | **0.66** | **0.86** |
| **Age** | **1.08** | **1.01** | **1.16** |
| **Gender** |  |  |  |
| Men | Ref | - | - |
| Women | 0.90 | 0.69 | 1.18 |
| **Education Level^b^** | **0.63** | **0.52** | **0.78** |
| **Area Deprivation Index^c^** | 1.01 | 0.93 | 1.08 |
| **Living Place** |  |  |  |
| With parents | Ref | - | - |
| Other | 0.84 | 0.65 | 1.08 |
| **Parents’ Highest Occupational Grade** |  |  |  |
| Home or other | Ref | - | - |
| Blue-collar worker, craftsman, farmer or employee | 0.54 | 0.15 | 1.95 |
| Intermediate worker | 0.49 | 0.13 | 1.86 |
| Executive, higher intellectual profession | 0.57 | 0.15 | 2.15 |
| **Depressive State^d^** |  |  |  |
| No | Ref | - | - |
| Yes | **2.10** | **1.57** | **2.82** |
| ^a^ Alcohol Use Disorders Identification Test to evaluate alcohol use  ^b^ Based on the International Standard Classification of Education  ^c^ Representing spatial socioeconomic disparities  ^d^ Measured using the Center of Epidemiologic Studies Depression scale (CESD) and a score ≥19 | | | |

Supplemental Table 7. Association between alcohol use and access to employment over a three-year of follow-up by differentiating between frequency of use and dependence and after adjusting for parents’ highest occupational grade (n=1,427).

| **Being unemployed over three -years of follow-up** | | | |
| --- | --- | --- | --- |
|  | **OR** | **95% CI** | |
| **Alcohol frequency of use^a^** |  |  |  |
| [0-2] | Ref | - | - |
| [3-5] | 0.84 | 0.60 | 1.17 |
| [6+] | 0.83 | 0.57 | 1.18 |
| **Alcohol dependence^b^** |  |  |  |
| [0] | Ref | - | - |
| [1-2] | 1.18 | 0.87 | 1.66 |
| [3+] | **1.66** | **1.17** | **2.35** |
| **Years of Follow-up** | **0.75** | **0.66** | **0.86** |
| **Age** | **1.08** | **1.01** | **1.16** |
| **Gender** |  |  |  |
| Men | Ref | - | - |
| Women | 0.87 | 0.67 | 1.14 |
| **Education Level^c^** | **0.63** | **0.52** | **0.78** |
| **Area Deprivation Index^d^** | 1.01 | 0.93 | 1.08 |
| **Living Place** |  |  |  |
| With parents | Ref | - | - |
| Other | 0.85 | 0.66 | 1.09 |
| **Parents’ Highest Occupational Grade** |  |  |  |
| Home or other | Ref | - | - |
| Blue-collar worker, craftsman, farmer or employee | 0.53 | 0.14 | 1.96 |
| Intermediate worker | 0.48 | 0.12 | 1.87 |
| Executive, higher intellectual profession | 0.56 | 0.14 | 2.16 |
| **Depressive State^e^** |  |  |  |
| No | Ref | - | - |
| Yes | **2.10** | **1.57** | **2.82** |
| ^a^ AUDIT sub-score for frequency of use by adding the scores of the first 3 items  ^b^ AUDIT sub-score for alcohol dependence by adding the score of items 4, 5, 6, 7, 8, 9 and 10  ^c^ Based on the International Standard Classification of Education  ^d^ Representing spatial socioeconomic disparities  ^e^ Measured using the Center of Epidemiologic Studies Depression scale (CESD) and a score ≥19 | | | |

Supplemental Table 8.Association between tobacco, cannabis and alcohol dependence and employment status over a three-year of follow-up (n=1,427).

| **Being unemployed over three -years of follow-up** | | | | | | | | | | |
| --- | --- | --- | --- | --- | --- | --- | --- | --- | --- | --- |
|  | **Model 1^a^** | | | **Model 2^b^** | | | **Model 3^c^** | | | |
|  | **OR** | **95% CI** | | **OR** | **95% CI** | | **OR** | **95% CI** | | |
| **Tobacco Use** |  |  |  |  |  |  |  |  |  | |
| Non-smoker | Ref | - | - | Ref | - | - | Ref | - | - | |
| Former smoker | 1.06 | 0.69 | 1.63 | 1.01 | 0.65 | 1.54 | 0.95 | 0.62 | 1.45 | |
| Current light smoker | 1.02 | 0.69 | 1.49 | 0.96 | 0.65 | 1.42 | 0.89 | 0.61 | 1.33 | |
| Current moderate smoker | 0.83 | 0.48 | 1.44 | 0.77 | 0.44 | 1.35 | 0.73 | 0.41 | 1.28 | |
| Current heavy smoker | 2.39 | 0.70 | 8.15 | 2.21 | 0.71 | 6.86 | 1.55 | 0.48 | 4.98 | |
| **Cannabis Consumption** |  |  |  |  |  |  |  |  |  | |
| Never | Ref | - | - | Ref | - | - | Ref | - | - | |
| Not during the previous year | 0.78 | 0.56 | 1.10 | 0.82 | 0.59 | 1.16 | 0.81 | 0.57 | 1.13 | |
| Less than once per month | 0.98 | 0.64 | 1.49 | 10.93 | 0.70 | 1.63 | 1.03 | 0.67 | 1.59 | |
| Less than once per week | 0.87 | 0.52 | 1.47 | 0.97 | 0.58 | 1.64 | 0.98 | 0.57 | 1.66 | |
| Once per week or more | **1.72** | **1.09** | **2.70** | **1.78** | **1.14** | **2.78** | **1.68** | **1.08** | **2.62** | |
| **Alcohol dependence^d^** |  |  |  |  |  |  |  |  |  | |
| [0] | Ref | - | - | Ref | - | - | Ref | - | - | |
| [1-2] | 1.13 | 0.84 | 1.53 | 1.14 | 0.84 | 1.54 | 1.12 | 0.83 | 1.52 | |
| [3+] | **1.50** | **1.06** | **2.13** | **1.52** | **1.08** | **2.15** | **1.49** | **1.05** | **2.11** | |
| ^a^ Univariate analysis adjusted to years of follow-up.  ^b^ Adjusted for sociodemographic factors  ^c^ Adjusted for sociodemographic factors and depressive state  ^d^ AUDIT sub-score for alcohol dependence by adding the score of items 4, 5, 6, 7, 8, 9 and 10 | | | | | | | | | |  |

Table 9. Association between tobacco use and employment status over three-year of follow-up (employment versus all the other forms of unemployment) (n=1,497).

| **Not being employed over three -years of follow-up** | | | | | | | | | |
| --- | --- | --- | --- | --- | --- | --- | --- | --- | --- |
|  | **Model 1^a^** | | | **Model 2^b^** | | | **Model 3^c^** | | |
|  | **OR** | **95% CI** | | **OR** | **95% CI** | | **OR** | **95% CI** | |
| **Tobacco Use** |  |  |  |  |  |  |  |  |  |
| Non-smoker | Ref | - | - | Ref | - | - | Ref | - | - |
| Former smoker | 1.19 | 0.84 | 1.69 | 1.19 | 0.84 | 1.69 | 1.11 | 0.78 | 1.57 |
| Current light smoker | 1.33 | 0.98 | 1.81 | 1.31 | 0.96 | 1.79 | 1.19 | 0.87 | 1.64 |
| Current moderate smoker | 0.99 | 0.62 | 1.59 | 0.94 | 0.58 | 1.52 | 0.86 | 0.53 | 1.41 |
| Current heavy smoker | 2.01 | 0.60 | 6.65 | 2.01 | 0.67 | 6.07 | 1.38 | 0.44 | 4.26 |
| **Years of Follow-up** | **0.77** | **0.69** | **0.87** | **0.77** | **0.69** | **0.87** | **0.78** | **0.69** | **0.87** |
| **Age** |  |  |  | 1.06 | 1.00 | 1.13 | 1.04 | 0.98 | 1.10 |
| **Gender** |  |  |  |  |  |  |  |  |  |
| Men |  |  |  | Ref | - | - | Ref | - | - |
| Women |  |  |  | 0.81 | 0.64 | 1.02 | **0.74** | **0.59** | **0.94** |
| **Education Level^d^** |  |  |  | **0.64** | **0.53** | **0.77** | **0.68** | **0.56** | **0.82** |
| **Area Deprivation Index^e^** |  |  |  | 1.01 | 0.94 | 1.07 | 1.00 | 0.93 | 1.07 |
| **Living Place** |  |  |  |  |  |  |  |  |  |
| With parents |  |  |  | Ref | - | - | Ref | - | - |
| Other |  |  |  | 0.94 | 0.75 | 1.18 | 0.95 | 0.75 | 1.19 |
| **Depressive State^f^** |  |  |  |  |  |  |  |  |  |
| No |  |  |  |  |  |  | Ref | - | - |
| Yes |  |  |  |  |  |  | **2.13** | **1.63** | **2.78** |
| ^a^ Univariate analysis adjusted to years of follow-up.  ^b^ Adjusted for sociodemographic factors  ^c^ Adjusted for sociodemographic factors and depressive state  ^d^ Based on the International Standard Classification of Education  ^e^ Measured using the Center of Epidemiologic Studies Depression scale (CESD) and a score ≥19  ^f^ Representing spatial socioeconomic disparities | | | | | | | | | |

Table 10. Association between cannabis use and employment status over three-year of follow-up (employment versus all the other forms of unemployment) (n=1,497).

| **Not being employed over three -years of follow-up** | | | | | | | | | |
| --- | --- | --- | --- | --- | --- | --- | --- | --- | --- |
|  | **Model 1^a^** | | | **Model 2^b^** | | | **Model 3^c^** | | |
|  | **OR** | **95% CI** | | **OR** | **95% CI** | | **OR** | **95% CI** | |
| **Cannabis Consumption** |  |  |  |  |  |  |  |  |  |
| Never used | Ref | - | - | Ref | - | - | Ref | - | - |
| Not during the previous year | 0.92 | 0.69 | 1.21 | 0.97 | 0.73 | 1.29 | 0.93 | 0.70 | 1.23 |
| At least once during the last 12 months but less than once a month | 1.21 | 0.87 | 1.69 | 1.26 | 0.90 | 1.75 | 1.19 | 0.84 | 1.66 |
| At least once a month but less than once per week | 1.15 | 0.76 | 1.75 | 1.20 | 0.79 | 1.83 | 1.13 | 0.74 | 1.75 |
| Once per week or more | **2.15** | **1.49** | **3.11** | **2.08** | **1.45** | **3.00** | **1.85** | **1.29** | **2.64** |
| **Years of Follow-up** | **0.77** | **0.69** | **0.87** | **0.78** | **0.69** | **0.87** | **0.78** | **0.69** | **0.88** |
| **Age** |  |  |  | **1.07** | **1.01** | **1.14** | 1.05 | 0.99 | 1.12 |
| **Gender** |  |  |  |  |  |  |  |  |  |
| Men |  |  |  | Ref | - | - | Ref | - | - |
| Women |  |  |  | 0.86 | 0.68 | 1.09 | **0.78** | **0.62** | **0.99** |
| **Education Level^d^** |  |  |  | **0.64** | **0.53** | **0.77** | **0.68** | **0.57** | **0.83** |
| **Area Deprivation Index^e^** |  |  |  | 1.01 | 0.94 | 1.08 | 1.01 | 0.94 | 1.07 |
| **Living Place** |  |  |  |  |  |  |  |  |  |
| With parents |  |  |  | Ref | - | - | Ref | - | - |
| Other |  |  |  | 0.92 | 0.73 | 1.15 | 0.93 | 0.74 | 1.17 |
| **Depressive State^f^** |  |  |  |  |  |  |  |  |  |
| No |  |  |  |  |  |  | Ref | - | - |
| Yes |  |  |  |  |  |  | **2.13** | **1.63** | **2.78** |
| ^a^ Univariate analysis adjusted to years of follow-up.  ^b^ Adjusted for sociodemographic factors  ^c^ Adjusted for sociodemographic factors and depressive state  ^d^ Based on the International Standard Classification of Education  ^e^ Measured using the Center of Epidemiologic Studies Depression scale (CESD) and a score ≥19  ^f^ Representing spatial socioeconomic disparities | | | | | | | | | |

Table 11. Association between alcohol use and employment status over three-year of follow-up (employment versus all the other forms of unemployment) (n=1,497).

| **Not being employed over three -years of follow-up** | | | | | | | | | |
| --- | --- | --- | --- | --- | --- | --- | --- | --- | --- |
|  | **Model 1^a^** | | | **Model 2^b^** | | | **Model 3^c^** | | |
|  | **OR** | **95% CI** | | **OR** | **95% CI** | | **OR** | **95% CI** | |
| **AUDIT^d^** |  |  |  |  |  |  |  |  |  |
| Low risk | Ref | - | - | Ref | - | - | Ref | - | - |
| No use | **1.76** | **1.09** | **2.82** | 1.54 | 0.95 | 2.48 | 1.44 | 0.89 | 2.33 |
| At risk | **1.39** | **1.09** | **1.78** | **1.39** | **1.08** | **1.78** | **1.34** | **1.04** | **1.71** |
| **Years of Follow-up** | **0.77** | **0.69** | **0.86** | **0.77** | **0.69** | **0.86** | **0.77** | **0.69** | **0.87** |
| **Age** |  |  |  | 1.06 | 0.99 | 1.12 | 1.04 | 0.98 | 1.10 |
| **Gender** |  |  |  |  |  |  |  |  |  |
| Men |  |  |  | Ref | - | - | Ref | - | - |
| Women |  |  |  | 0.86 | 0.68 | 1.09 | 0.79 | 0.62 | 1.01 |
| **Education Level^e^** |  |  |  | **0.64** | **0.53** | **0.78** | **0.68** | **0.57** | **0.83** |
| **Area deprivation index^f^** |  |  |  | 1.01 | 0.94 | 1.07 | 1.01 | 0.93 | 1.07 |
| **Living Place** |  |  |  |  |  |  |  |  |  |
| With parents |  |  |  | Ref | - | - | Ref | - | - |
| Other |  |  |  | 0.93 | 0.74 | 1.16 | 0.94 | 0.75 | 1.18 |
| **Depressive State^g^** |  |  |  |  |  |  |  |  |  |
| No |  |  |  |  |  |  | Ref | - | - |
| Yes |  |  |  |  |  |  | **2.12** | **1.63** | **2.75** |
| ^a^ Univariate analysis adjusted to years of follow-up.  ^b^ Adjusted for sociodemographic factors  ^c^ Adjusted for sociodemographic factors and depressive state  ^d^ Alcohol Use Disorders Identification Test to evaluate alcohol use  ^e^ Based on the International Standard Classification of Education  ^f^ Measured using the Center of Epidemiologic Studies Depression scale (CESD) and a score ≥19  ^g^ Representing spatial socioeconomic disparities | | | | | | | | | |

Table 12. Association between alcohol use and employment status over three-year of follow-up (employment versus all the other forms of unemployment) by differentiating between frequency of use and dependence (n=1,497).

| **Not being employed over three -years of follow-up** | | | | | | | | | |
| --- | --- | --- | --- | --- | --- | --- | --- | --- | --- |
|  | **Model 1^a^** | | | **Model 2^b^** | | | **Model 3^c^** | | |
|  | **OR** | **95% CI** | | **OR** | **95% CI** | | **OR** | **95% CI** | |
| **Alcohol frequency of use^d^** |  |  |  |  |  |  |  |  |  |
| [0-2] | Ref | - | - | Ref | - | - | Ref | - | - |
| [3-5] | 0.86 | 0.65 | 1.14 | 0.91 | 0.68 | 1.21 | 0.88 | 0.66 | 1.17 |
| [6+] | 0.85 | 0.62 | 1.17 | 0.92 | 0.66 | 1.26 | 0.92 | 0.66 | 1.26 |
| **Alcohol dependence^e^** |  |  |  |  |  |  |  |  |  |
| [0] | Ref | - | - | Ref | - | - | Ref | - | - |
| [1-2] | 1.19 | 0.91 | 1.57 | 1.16 | 0.88 | 1.52 | 1.12 | 0.85 | 1.48 |
| [3+] | **1.66** | **1.20** | **2.30** | **1.61** | **1.17** | **2.22** | **1.51** | **1.10** | **2.07** |
| **Years of follow-up** | **0.78** | **0.69** | **0.87** | **0.78** | **0.69** | **0.87** | **0.78** | **0.70** | **1.13** |
| **Age** |  |  |  | 1.06 | 1.01 | 1.13 | 1.04 | 0.98 | 1.10 |
| **Gender** |  |  |  |  |  |  |  |  |  |
| Men |  |  |  | Ref | - | - | Ref | - | - |
| Women |  |  |  | 0.84 | 0.66 | 1.07 | **0.77** | **0.61** | **0.98** |
| **Education Level^f^** |  |  |  | **0.63** | **0.53** | **0.77** | **0.68** | **0.56** | **0.82** |
| **Area deprivation index^g^** |  |  |  | 1.01 | 0.94 | 1.07 | 1.01 | 0.93 | 1.07 |
| **Living Place** |  |  |  |  |  |  |  |  |  |
| With parents |  |  |  | Ref | - | - | Ref | - | - |
| Other |  |  |  | 0.94 | 0.75 | 1.17 | 0.95 | 0.75 | 1.19 |
| **Depressive State^h^** |  |  |  |  |  |  |  |  |  |
| No |  |  |  |  |  |  | Ref | - | - |
| Yes |  |  |  |  |  |  | **2.12** | **1.63** | **2.75** |
| ^a^ Univariate analysis adjusted to years of follow-up.  ^b^ Adjusted for sociodemographic factors  ^c^ Adjusted for sociodemographic factors and depressive state  ^d^ AUDIT sub-score for frequency of use by adding the scores of the first 3 items  ^e^ AUDIT sub-score for alcohol dependence by adding the score of items 4, 5, 6, 7, 8, 9 and 10  ^f^ Based on the International Standard Classification of Education  ^g^ Representing spatial socioeconomic disparities  ^h^ Measured using the Center of Epidemiologic Studies Depression scale (CESD) and a score ≥19 | | | | | | | | | |

Supplemental Table 13. Association between cannabis use and employment status over a three-years of follow-up after excluding participants with psychiatric history (n=1,324).

| **Being unemployed over three -years of follow-up** | | | | | | | | | |
| --- | --- | --- | --- | --- | --- | --- | --- | --- | --- |
|  | **Model 1^a^** | | | **Model 2^b^** | | | **Model 3^c^** | | |
|  | **OR** | **95% CI** | | **OR** | **95% CI** | | **OR** | **95% CI** | |
| **Cannabis Consumption** |  |  |  |  |  |  |  |  |  |
| Never used | Ref | - | - | Ref | - | - | Ref | - | - |
| Not during the previous year | 0.80 | 0.57 | 1.12 | 0.83 | 0.59 | 1.16 | 0.81 | 0.58 | 1.15 |
| At least once during the last 12 months but less than once a month | 1.13 | 0.77 | 1.68 | 1.19 | 0.80 | 1.77 | 1.13 | 0.75 | 1.68 |
| At least once a month but less than once per week | 0.92 | 0.56 | 1.53 | 0.97 | 0.58 | 1.61 | 0.93 | 0.55 | 1.56 |
| Once per week or more | **1.78** | **1.15** | **2.75** | **1.74** | **1.12** | **2.68** | **1.59** | **1.03** | **2.46** |
| ^a^ Univariate analysis adjusted to years of follow-up.  ^b^ Adjusted for sociodemographic factors  ^c^ Adjusted for sociodemographic factors and depressive state | | | | | | | | | |

Supplemental Table 14. Association between alcohol use and employment status over a three-years of follow-up after excluding participants with psychiatric history (n=1,324).

| **Being unemployed over three -years of follow-up** | | | | | | | | | |
| --- | --- | --- | --- | --- | --- | --- | --- | --- | --- |
|  | **Model 1^a^** | | | **Model 2^b^** | | | **Model 3^c^** | | |
|  | **OR** | **95% CI** | | **OR** | **95% CI** | | **OR** | **95% CI** | |
| **Alcohol dependence^d^** |  |  |  |  |  |  |  |  |  |
| [0] | Ref | - | - | Ref | - | - | Ref | - | - |
| [1-2] | **1.87** | **1.14** | **3.08** | 1.67 | 1.01 | 2.76 | 1.55 | 0.94 | 2.56 |
| [3+] | **1.39** | **1.07** | **1.79** | **1.37** | **1.05** | **1.79** | **1.33** | **1.02** | **1.80** |
| ^a^ Univariate analysis adjusted to years of follow-up.  ^b^ Adjusted for sociodemographic factors  ^c^ Adjusted for sociodemographic factors and depressive state  ^d^ AUDIT sub-score for alcohol dependence by adding the score of items 4, 5, 6, 7, 8, 9 and 10 | | | | | | | | | |

Supplemental Table 15. Association between alcohol use and employment status over 3-years follow-up after excluding participants who initiated alcohol use before the age of 13 (n=1,329).

|  | **Being unemployed over 3 years of follow-up** | | | | | | | | | | | |
| --- | --- | --- | --- | --- | --- | --- | --- | --- | --- | --- | --- | --- |
|  | **Model 1^a^** | | | | **Model 2^b^** | | | | **Model 3^c^** | | | |
|  | **OR** | **95% CI** | | **P** | **OR** | **95% CI** | | **P** | **OR** | **95% CI** | | **P** |
| **Alcohol dependence^d^** |  |  |  |  |  |  |  |  |  |  |  |  |
| [0] | Ref | - | - | - | Ref | - | - | - | Ref | - | - | - |
| [1-2] | 1.21 | 0.87 | 1.68 | 0.257 | 1.19 | 0.86 | 1.66 | 0.286 | 1.15 | 0.83 | 1.61 | 0.391 |
| [3+] | **1.89** | **1.32** | **2.71** | **0.001** | **1.87** | **1.30** | **2.68** | **0.001** | **1.79** | **1.24** | **2.57** | **0.002** |
| ^a^ Univariate analysis adjusted to years of follow-up.  ^b^ Adjusted for sociodemographic factors  ^c^ Adjusted for sociodemographic factors and depressive state  ^d^ AUDIT sub-score for alcohol dependence by adding the score of items 4, 5, 6, 7, 8, 9 and 10 | | | | | | | | | | | | |
